# Integrated Assessment of Stress Hyperglycemia and Glycemic Variability for Mortality Prediction in Non-St-Segment Elevation Myocardial Infarction and St-Segment Elevation Myocardial Infarction: A Glycometabolic Phenotype-Stratified Analysis

**DOI:** 10.31083/RCM50449

**Published:** 2026-08-24

**Authors:** Jinlin Hu, Jingyu Bo, Kunyang He, Liangbing Yang, Miao Zhang, Teng Ge, Bo Ning, Guanmou Li, Rongjun Zou, Xiaoping Fan

**Affiliations:** ^1^Department of Cardiac and Great Vascular Surgery, The Second Affiliated Hospital of Guangzhou University of Chinese Medicine (Guangdong Provincial Hospital of Chinese Medicine), 510120 Guangzhou, Guangdong, China; ^2^Guangdong Provincial Key Laboratory of TCM Emergency Research, 510120 Guangzhou, Guangdong, China; ^3^Zhu Jiang Hospital, Southern Medical University, 510260 Guangzhou, Guangdong, China

**Keywords:** NSTEMI, STEMI, stress hyperglycemia ratio, glycemic variability, MIMIC-IV database

## Abstract

**Background:**

Precise risk stratification in myocardial infarction (MI) remains challenging, particularly when integrating acute dysglycemia with chronic metabolic status. Prior evaluations of stress hyperglycemia ratio (SHR) and glycemic variability (GluCV) have seldom accounted for underlying glucose metabolic phenotypes, potentially obscuring their prognostic utility in distinct MI subtypes.

**Methods:**

In this retrospective cohort study, we identified 3628 non-ST-segment elevation myocardial infarction (NSTEMI) and 825 ST-segment elevation myocardial infarction (STEMI) patients from the Medical Information Mart for Intensive Care IV (MIMIC-IV) database. Participants were stratified by glycemic status: normal glucose regulation (NGR), pre-diabetes (Pre-DM), and diabetes mellitus (DM). We assessed the individual and combined prognostic value of SHR and GluCV for one-year all-cause mortality. Machine learning (ML) models were subsequently developed to optimize risk prediction.

**Results:**

The combination of SHR and GluCV effectively stratified mortality risk across all glycemic phenotypes in both NSTEMI and STEMI patients. Notably, within NSTEMI cohorts, the subgroup characterized by both high GluCV and low SHR exhibited the poorest survival. In comparative analyses, GluCV demonstrated superior prognostic performance over SHR for NSTEMI outcomes. Among five ML algorithms tested, the Random Forest model achieved the highest predictive accuracy [NGR (area under the curve (AUC): 0.885, 95% confidence interval (CI): 0.882–0.888), Pre-DM (AUC: 0.854, 95% CI: 0.85–0.857), DM (AUC: 0.87, 95% CI: 0.868–0.871)], with GluCV consistently identified as a top-ranking feature.

**Conclusions:**

By concurrently evaluating SHR and GluCV within a glycemic stratification framework, this study refines mortality prediction in MI patients. Our findings advocate for a shift from uniform glycemic targets to phenotype-specific management strategies. The developed ML tool offers a practical approach for individualized risk assessment in clinical practice.

## 1. Introduction

Acute Coronary Syndromes (ACS), encompassing ST-segment elevation myocardial infarction (STEMI) and non-ST-segment elevation myocardial infarction (NSTEMI), represent critical manifestations of coronary artery disease with differing pathophysiological and therapeutic implications. While both involve cardiomyocyte necrosis, their management—particularly regarding the timing of revascularization—diverges significantly, underscoring the need for phenotype-specific risk stratification tools [1]. Complicating this landscape is the profound heterogeneity introduced by dysglycemia. Diabetes is a well-established disease associated with increased cardiovascular risk, yet emerging evidence suggests that acute glycemic disturbances, even in normoglycemic individuals, may exert substantial prognostic influence [2,3]. However, existing risk stratification frameworks for myocardial infarction (MI) have largely failed to integrate acute glycemic metrics with chronic glucose metabolic phenotypes, and few studies have distinguished the prognostic value of these metrics between NSTEMI and STEMI—two MI subtypes with distinct clinical trajectories and pathophysiological drivers.

Two key metrics capturing acute dysglycemia are the stress hyperglycemia ratio (SHR) and glycemic variability (GluCV). SHR quantifies the relative hyperglycemic response during acute illness by comparing admission blood glucose to chronic glycemic levels derived from HbA1c [4,5], whereas GluCV measures the amplitude of glucose fluctuations over time, which is associated with oxidative stress and endothelial dysfunction [6]. Although both SHR and GluCV have been associated with adverse outcomes in broad cardiovascular populations [7,8], three critical gaps remain in the current literature: (1) prior evaluations have seldom stratified patients by pre-existing glycemic status (normal glucose regulation [NGR], pre-diabetes [Pre-DM], diabetes mellitus [DM]) when assessing SHR and GluCV, potentially obscuring their prognostic utility in distinct metabolic subgroups; (2) the differential impact of SHR and GluCV on mortality risk between NSTEMI and STEMI has not been rigorously characterized, despite the fundamental pathophysiological differences between these two subtypes; and (3) the synergistic prognostic value of combined SHR and GluCV profiling—and the potential identification of novel high-risk phenotypic subgroups—remains unexplored in MI patients stratified by both metabolic status and the type of infarction [9,10].

This study investigates the prognostic value of SHR and GluCV for 1-year mortality in NSTEMI and STEMI patients from the Medical Information Mart for Intensive Care IV (MIMIC-IV) database, stratified by baseline glycemic status (NGR, Pre-DM, DM). We hypothesize that this dual stratification (glycemic phenotype + MI subtype) will reveal distinct high-risk profiles, particularly highlighting GluCV as a dominant prognosticator in NSTEMI. Furthermore, we develop machine learning (ML)-based prediction models for NSTEMI to translate these findings into clinical practice. This approach aims to shift MI care from uniform targets toward personalized glycemic management.

## 2. Materials and Methods

### 2.1 Data Source and Study Population

This observational analysis utilized data from the MIMIC-IV v3.1, a publicly available repository of de-identified health records from patients admitted to intensive care units at a tertiary care center. The use of this database was authorized following completion of the required Collaborative Institutional Training Initiative (CITI) training (Certification ID: 49,270,219). Given the de-identified nature of the data, informed consent was waived for this study.

**Fig. 1.**
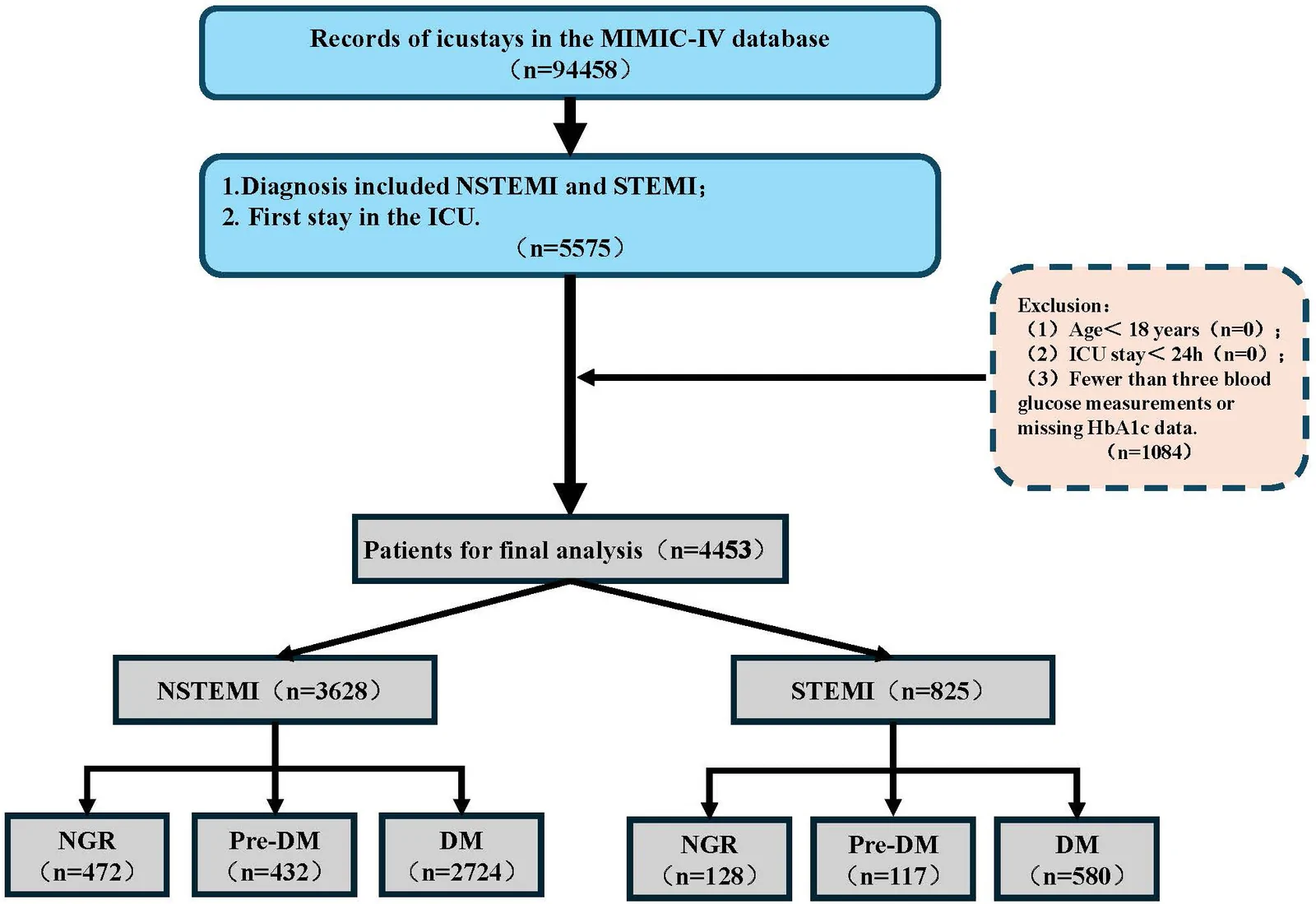
**Patient screening flowchart of the Medical Information Mart for Intensive Care IV (MIMIC-IV) database**. NSTEMI, non-ST-segment elevation myocardial infarction; STEMI, ST-segment elevation myocardial infarction; ICU, intensive care unit; NGR, normal glucose regulation; Pre-DM, pre-diabetes; DM, diabetes mellitus.

Adult patients (≥18 years) with a primary hospital discharge diagnosis of either NSTEMI or STEMI, based on International Classification of Diseases-10 (ICD-10) codes (**Supplementary Table 1**), were initially screened. To ensure data quality and relevance for glycemic analysis, we excluded patients with an intensive care unit (ICU) length of stay less than 24 hours, those with fewer than three blood glucose measurements during their ICU stay, and those missing HbA1c values. For subjects with multiple admissions, only the first eligible ICU stay was included to maintain independence of observations. The detailed patient selection process is outlined in Fig. 1.

### 2.2 Data Extraction and Key Variable Definitions

Clinical data were extracted using Structured Query Language (SQL). Extracted variables encompassed:

Demographic information (age, sex, race).

Severity-of-illness scores [e.g., Acute Physiology and Chronic Health Evaluation III (APACHE III), Sequential Organ Failure Assessment (SOFA)] assessed at ICU admission.

Vital signs and laboratory parameters from the first 24 hours of ICU stay.

Comorbidities and treatment details (e.g., history of diabetes, use of insulin, mechanical ventilation).

All serial blood glucose measurements during the ICU stay.

### 2.3 Definition of Exposure Variables

SHR: Calculated as: SHR = Admission Blood Glucose (mg/dL) / [ (28.7 × HbA1c (%)) – 46.7].

GluCV: Expressed as the coefficient of variation (CV), calculated as (Standard Deviation of all glucose measurements / Mean of all glucose measurements) × 100%. Glycemic Status Stratification: Patients were categorized into three groups:

NGR: HbA1c <5.7% and no prior diagnosis of diabetes; Pre-DM: HbA1c 5.7–6.4% and no prior diagnosis of diabetes; DM: HbA1c ≥6.5% or a documented history of diabetes.

Variables with ≥20% missing values were excluded to reduce potential bias, while variables with <20% missing values were imputed using multiple imputation (**Supplementary Fig. 1**).

### 2.4 Outcome Measures

The main outcome of this study was 1-year all-cause mortality.

### 2.5 Statistical Analysis

Based on the tertiles of SHR and GluCV in the NSTEMI patients (SHR: <0.85, 0.85–1.11, >1.11; GluCV: <24.88, 24.88–37.61, >37.61), and the tertiles of SHR and GluCV in the STEMI patients (SHR: <0.92, 0.92–1.25, >1.25; GV: <22.96, 22.96–33.65, >33.65), participants were classified into high and low groups. The top tertile was regarded as “high”, and the bottom tertile was regarded as “low”. The normality of continuous variables was assessed using the Shapiro-Wilk test; among these, normally distributed variables (expressed as mean ± standard deviation [SD]) were analyzed via Student’s *t*-test or one-way analysis of variance (ANOVA), while non-normally distributed variables (expressed as median [interquartile range, IQR]) were compared using the Wilcoxon rank-sum test. Categorical variables, presented as counts (percentages), were evaluated using the χ^2^ test or Fisher’s exact test, as appropriate.

Multicollinearity among candidate variables was assessed using the variance inflation factor (VIF), and variables with a VIF >5 were excluded from subsequent multivariable models. Variable selection was performed using the least absolute shrinkage and selection operator (LASSO) regression to remove redundant predictors; the coefficient trajectories and cross‑validation for optimal lambda are presented in **Supplementary Fig. 2**. To estimate the cumulative risk of all-cause mortality, Kaplan-Meier (KM) survival curves were generated. Concurrently, Cox proportional hazards regression models were conducted in three sequential stages: Model 1 (unadjusted), Model 2 (adjusted for age and glycated hemoglobin [HbA1c]), and Model 3 (adjusted for all potential covariates). The selection of covariates was based on two criteria: (1) clinically meaningful indicators and (2) variables that showed statistical significance in univariate analysis; in addition, collinearity was strictly controlled (VIF <5) during model construction.

Restricted cubic splines (RCS) were employed to explore the dose-response relationships between SHR and GluCV. The proportional hazards assumption of Cox models was verified using Schoenfeld residuals. Landmark analyses were performed to assess temporal changes in the mortality risk profile, and receiver operating characteristic (ROC) curves were constructed to compare the predictive performance (including area under the curve [AUC], sensitivity, and specificity) of different metrics. Subgroup analyses, stratified by age, body mass index (BMI), comorbidities, invasive procedures, and mechanical ventilation status, were depicted using forest plots, and hazard ratios (HRs) and 95% confidence intervals (CIs).

For ML-based prediction of 1-year all-cause mortality, the Boruta algorithm was first utilized to rank the importance of candidate features. Subsequently, the dataset was subjected to random division into a training subset (70%) and a testing subset (30%) for model development and validation. Five models—support vector machine (SVM), adaptive boosting (Adaboost), random forest (RF), extreme gradient boosting (XGBoost), and light gradient-boosted machine (LightGBM)—were developed using selected features. All analyses were performed in R software (version 4.5.1, Vienna, Austria), with two-tailed *p* values < 0.05 considered statistically significant.

## 3. Results

### 3.1 Clinic Baseline Characteristics

A total of 3628 patients with NSTEMI and 825 patients with STEMI were identified in this study. Baseline characteristics of NSTEMI patients are presented in **Supplementary Table 2**, while those of STEMI patients are shown in **Supplementary Table 3**. Results of univariate Cox regression analyses are available in **Supplementary Tables 4,5**. VIF for NSTEMI and STEMI patients are displayed in **Supplementary Tables 6,7**, respectively, indicating the absence of multicollinearity among variables.

Overall, the median age of NSTEMI patients (71 years) was higher than that of STEMI patients (68 years). Although the first blood glucose measurement and SHR were lower in NSTEMI patients than in STEMI patients, NSTEMI patients exhibited higher HbA1c levels and GluCV during their ICU stay. Notably, the 1-year survival rate was also lower in NSTEMI patients (72.52%) compared with STEMI patients (73.21%). NSTEMI patients had more frequent invasive catheter placements and higher insulin infusion requirements than STEMI patients. Additionally, NSTEMI patients showed higher levels of Acute Physiology Score III (APSIII), Logistic Organ Dysfunction System (LODS), Oxford acute severity of illness score (OASIS), Simplified Acute Physiology Score II (SAPSII), creatinine, Albumin (Alb), and Blood Urea Nitrogen (BUN). In contrast, STEMI patients had higher levels of lactate, Creatine Kinase‑MB (CK-MB), Hemoglobin(Hb), platelets, White Blood Cell (WBC), and Red Blood Cell (RBC) than NSTEMI patients.

### 3.2 The Association Between GluCV and Mortality in Patients With NSTEMI and STEMI

**Fig. 2.**
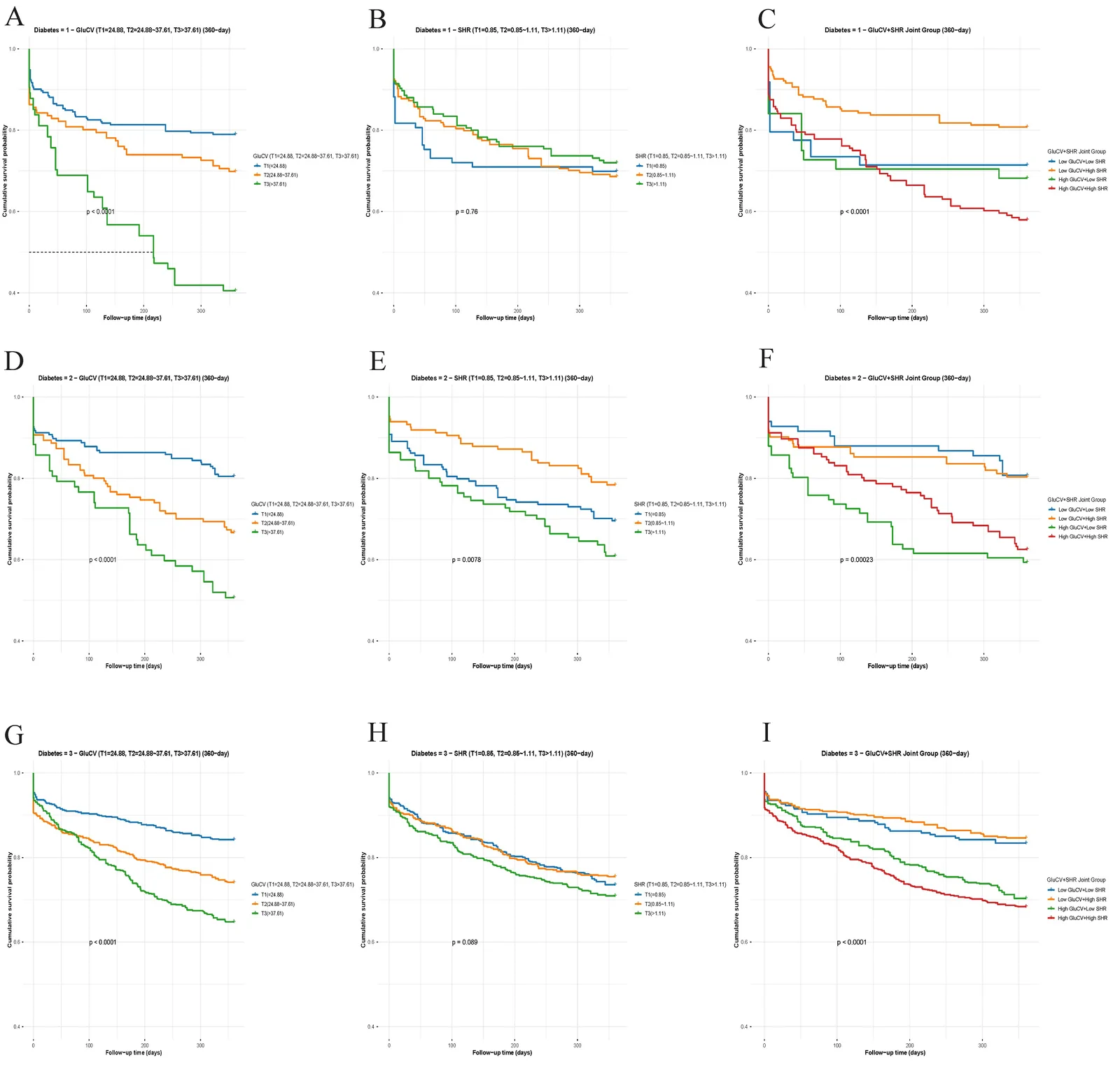
**The Kaplan-Meier curves for the 1-year mortality of stress hyperglycemia ratio (SHR), GluCV, and their combinations in NSTEMI patients**. (A) Glycemic variability (GluCV) of normal glucose regulation (NGR); (B) SHR of NGR; (C) GluCV+SHR of NGR; (D) GluCV of Pre-DM; (E) SHR of Pre-DM; (F) GluCV+SHR of Pre-DM; (G) GluCV of DM; (H) SHR of DM; (I) GluCV+SHR of DM. [(A,D,G) T1 GluCV <24.88; T2 24.88 ≤ GluCV ≤ 37.61; T3 GluCV >37.61. (B,E,H) T1 SHR <0.85; T2 0.85 ≤ SHR ≤ 1.11; T3 SHR >1.11. (C,F,I) Group 1: Low SHR and Low GluCV (SHR <0.85 and GluCV <24.88); Group 2: Low SHR and High GluCV (SHR <0.85 and GluCV >37.61); Group 3: High SHR and Low GluCV (SHR >1.11 and GluCV <24.88); Group 4: High SHR and High GluCV (SHR >1.11 and GluCV >37.61)].

**Fig. 3.**
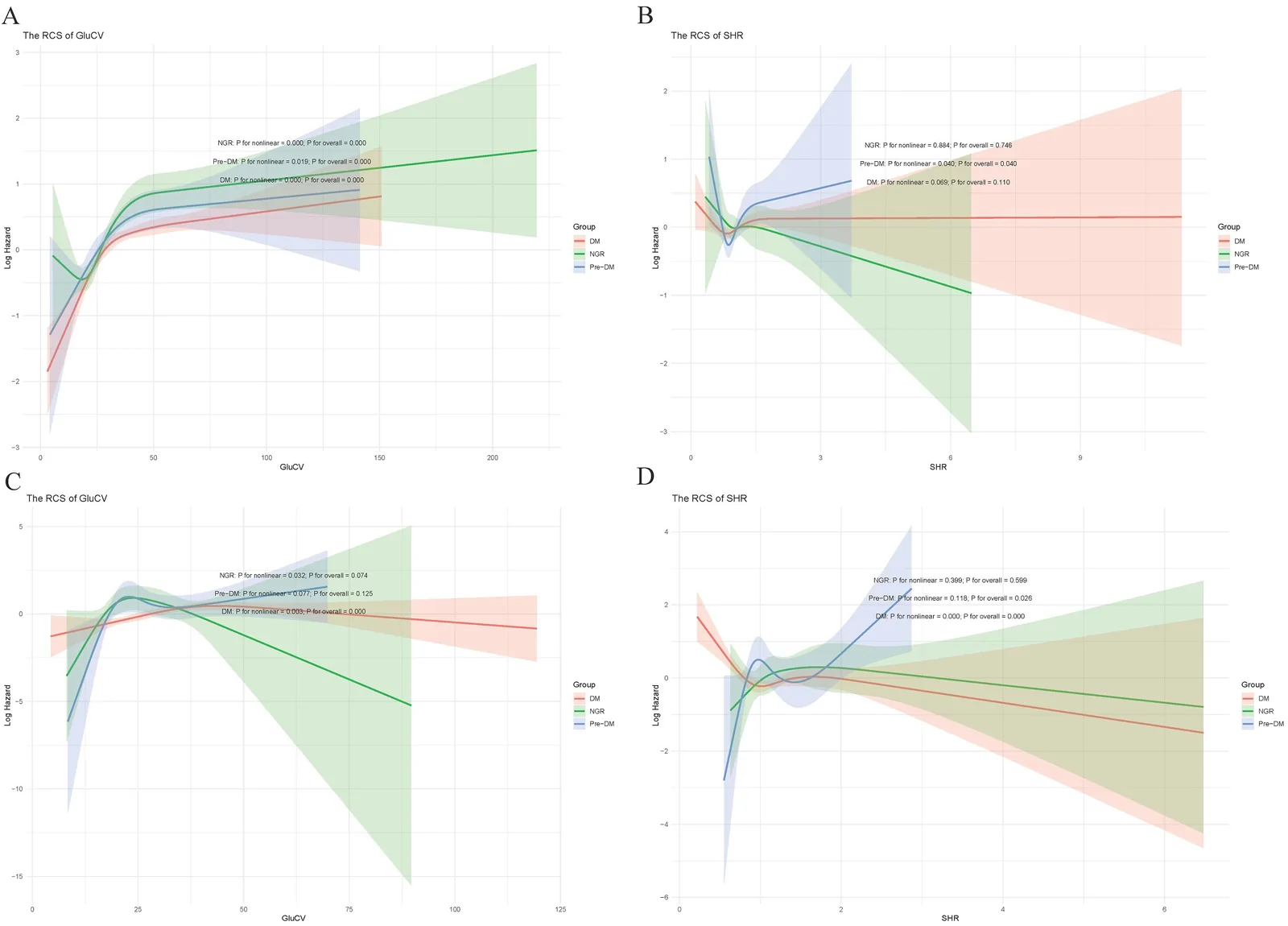
**Multivariable-adjusted restricted cubic spline analyses of SHR and GluCV for 1-year mortality**. (A) The restricted cubic splines (RCS) of GluCV in patients with NSTEMI. (B) The RCS of SHR in patients with NSTEMI. (C) The RCS of GluCV in patients with STEMI. (D) The RCS of SHR in patients with STEMI.

The association between GluCV and 1-year mortality in NSTEMI patients is illustrated in Fig. 2A,D,G. Tertiles of GluCV showed nearly identical trends across NGR, Pre-DM, and DM subgroups, with the highest tertile consistently associated with significantly increased mortality. A significant association between GluCV and 1-year mortality was observed in the overall population and all glycemic subgroups, except for the highest GluCV tertile in the Pre-DM subgroup, where the increase in 1-year mortality was not statistically significant (HR 1.31, 95% CI: 0.60–2.86, *p* = 0.5; **Supplementary Table 8**). RCS analysis revealed a significant non-linear dose-response relationship between GluCV and clinical outcomes across all glucose metabolic subgroups (NGR: *p* for non-linearity = 0.000; Pre-DM: *p* for non-linearity = 0.019; DM: *p* for non-linearity = 0.000; Fig. 3A). Subgroup analysis demonstrated that GluCV (**Supplementary Fig. 3**) was consistently and significantly associated with adverse outcomes in NSTEMI patients across most subgroups, except for the “no ventilation” subgroup (HR: 1.00, 95% CI: 0.99–1.01, *p* = 0.73).

In contrast to NSTEMI patients, STEMI patients in the NGR and Pre-DM subgroups showed no significant differences in mortality across SHR tertiles; only STEMI patients with DM in the highest SHR tertile had significantly increased mortality (**Supplementary Fig. 4B,E,H**). In the overall population, the NGR subgroup, and the DM subgroup of STEMI patients, the middle SHR tertile was significantly associated with increased 1-year mortality (**Supplementary Table 9**). RCS analysis indicated a significant non-linear association between GluCV and clinical outcomes in the DM subgroup of STEMI patients (DM: *p* for non-linearity = 0.003), while no significant associations were observed in the NGR or Pre-DM subgroups (Fig. 3C).

STEMI patients exhibited significant interactions between GluCV (**Supplementary Fig. 4A,D,G**) and mortality in four subgroups (chronic pulmonary disease, invasive procedures, ventilation mode, and metabolic status) (**Supplementary Fig. 5**). Interestingly, besides patients who underwent multiple invasive procedures (HR: 1.06, 95% CI: 1.02–1.10, *p* = 0.005) or had diabetes (HR: 1.02, 95% CI: 1.01–1.03, *p* = 0.004), GluCV was also significantly associated with mortality risk in STEMI patients without chronic pulmonary disease (HR: 1.02, 95% CI: 1.01–1.03, *p* < 0.001) and those receiving non-invasive ventilation (HR: 1.03, 95% CI: 1.01–1.04, *p* < 0.001).

### 3.3 The Association Between SHR and Mortality in Patients With NSTEMI and STEMI

In NSTEMI patients, no significant differences in mortality across SHR tertiles were observed in the NGR and DM subgroups, whereas in the Pre-DM subgroup, mortality was higher in both the lowest and highest SHR tertiles than in the middle tertile (Fig. 2B,E,H). In the fully adjusted Cox proportional hazards regression analysis of the entire NSTEMI cohort, patients in the highest SHR tertile had a 0.97-fold increased risk of 1-year all-cause mortality compared with those in the lowest tertile (HR: 0.97, 95% CI: 0.93–1.00). Stratified analysis by glucose metabolic subgroups showed consistent associations between SHR and mortality; however, the increase in 1-year risk was not statistically significant in the Pre-DM (HR: 1.63, 95% CI: 0.77–3.49) and the DM (HR: 1.31, 95% CI: 0.60–2.86) subgroups (**Supplementary Table 9**). RCS analysis revealed a non-linear association between SHR and 1-year mortality in the Pre-DM subgroup (*p* for non-linearity = 0.04), whereas no significant dose-response associations were observed between SHR and mortality in the NGR or DM subgroups (Fig. 3B). However, subgroup analysis (**Supplementary Fig. 6**) showed that SHR had no significant effect on 1-year mortality in NSTEMI patients with a different metabolic status. Instead, SHR was significantly associated with 1-year mortality in NSTEMI patients with chronic pulmonary disease or multiple invasive catheter placements. After further adjustment for insulin therapy within 48 hours of ICU admission, the high SHR+low GluCV phenotype remained an independent predictor of 1‑year mortality, confirming that this phenotype is not an artifact of intensive insulin therapy (**Supplementary Tables 10,11**).

Compared with NSTEMI patients, STEMI patients in the NGR and Pre-DM subgroups exhibited the same pattern (no significant SHR-mortality association), whereas STEMI patients with DM in the lowest SHR tertile had significantly increased mortality (**Supplementary Fig. 4B,E,H**). In fully adjusted Cox proportional hazards regression analysis of the entire STEMI cohort, patients in the highest SHR tertile had a 0.98-fold increased risk of 1-year all-cause mortality compared with those in the lowest tertile (HR: 0.98, 95% CI: 0.77–1.26). Stratified analysis according to glucose metabolic subgroups confirmed consistent associations between SHR and mortality in STEMI patients, whereas significant differences were observed only in the middle tertile of the NGR and DM subgroups (**Supplementary Table 9**). RCS analysis revealed distinctly different effects of SHR on STEMI risk across glucose statuses (Fig. 3D). A linear dose-response relationship was observed between SHR and 1-year mortality in the Pre-DM subgroup (*p* for non-linearity = 0.118), while a significant non-linear association was noted in the DM subgroup (*p* = 0.000). No association between SHR and 1-year mortality was found in the NGR subgroup. Subgroup analysis (**Supplementary Fig. 7**) showed that SHR was significantly associated with 1-year mortality in the DM subgroup of STEMI patients. Additionally, SHR was significantly associated with 1-year mortality in STEMI patients with HbA1c ≥6.5%, multiple invasive catheter placements, or mechanical ventilation.

### 3.4 The Association of Combined SHR and GluCV With Mortality in Patients With NSTEMI and STEMI

K-M curves depicting 1-year mortality according to the combined SHR and GluCV status in NSTEMI patients are shown in Fig. 2C,F,I. Across all glucose metabolic subgroups of NSTEMI patients, the “high GluCV + low SHR” subgroup had the highest mortality risk. K-M curves for STEMI patients are presented in **Supplementary Fig. 4C,F,I**. In the NGR and DM subgroups of STEMI patients, the “high SHR + low GluCV” subgroup also had the highest mortality risk (**Supplementary Table 12**); however, no statistically significant association with mortality was observed in the Pre-DM subgroup.

**Fig. 4.**
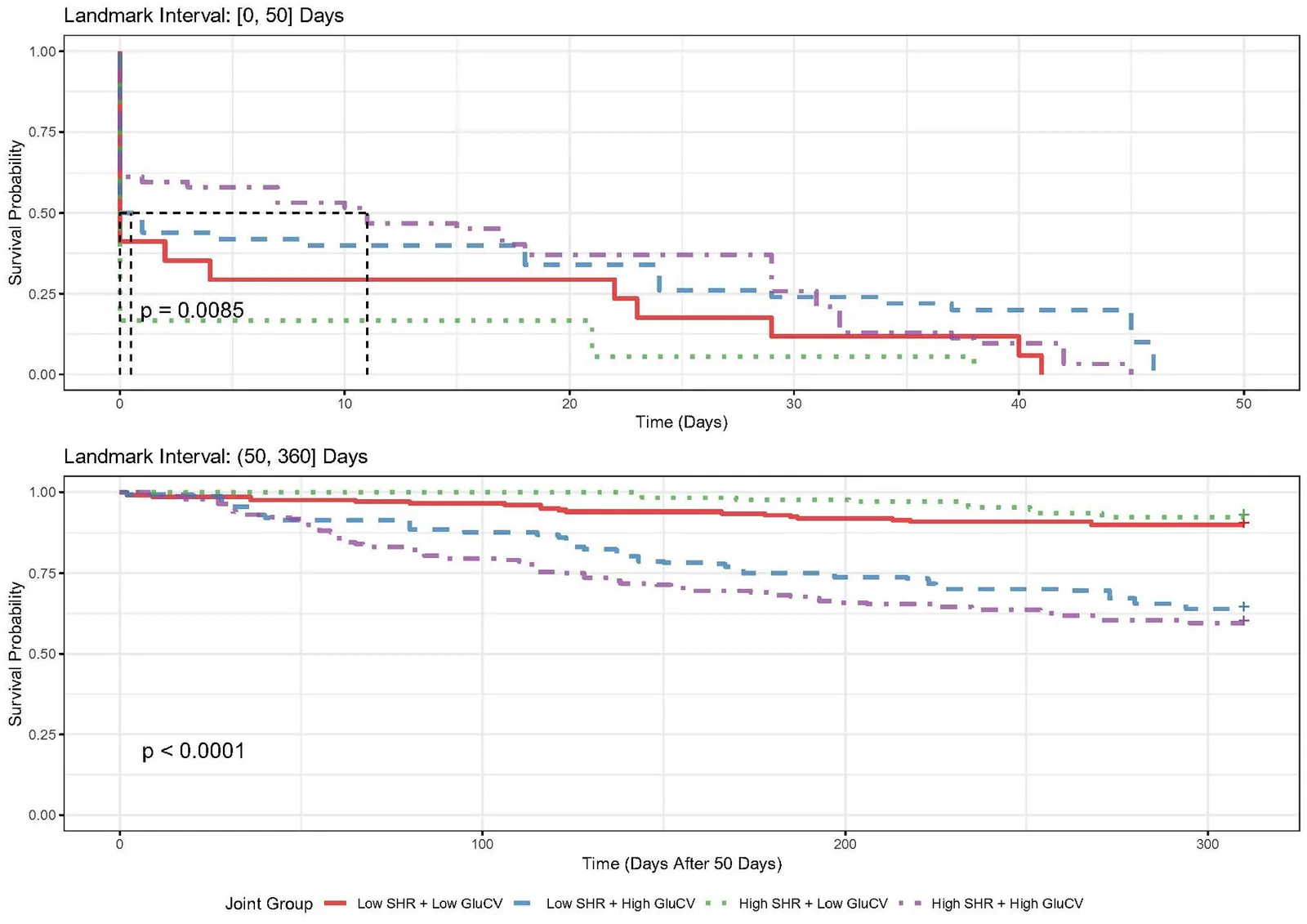
**Landmark survival analysis of 1-year mortality with combined SHR and GluCV assessment in DM of NSTEMI patients (The 200th day serves as the dividing point)**.

Proportional hazards tests for NSTEMI patients showed constant HRs between combined SHR/GluCV status and mortality over time in the NGR and Pre-DM subgroups [NGR: *p* = 0.092 (**Supplementary Table 13**); Pre-DM: *p* = 0.105 (**Supplementary Table 14**)]. A non-constant HR was observed in the DM subgroup (*p* = 0.021, **Supplementary Table 15**), with beta coefficients showing obvious fluctuations around day 200 (**Supplementary Fig. 8**). Further analysis revealed no significant difference in survival among DM patients within 200 days after discharge (*p* = 0.17); however, survival differences became significant after day 200 (*p* < 0.001). Patients in the “low SHR + low GluCV” subgroup had the highest 1-year survival rate compared with other combinations (Fig. 4).

In proportional hazards tests for STEMI patients, constant HRs between combined SHR/GluCV status and mortality were observed in the NGR and DM subgroups [NGR: *p* = 0.758 (**Supplementary Table 16**); DM: *p* = 0.116 (**Supplementary Table 10**)], while a non-constant HR was noted in the Pre-DM subgroup (*p* = 0.044, **Supplementary Table 17**), with beta coefficients decreasing continuously and then gradually increasing after day 50 (**Supplementary Fig. 9**). As shown in **Supplementary Fig. 10**, Pre-DM patients did not exhibit a significant survival difference during the first 50 days after discharge (*p* = 0.17). A significant differences in survival emerged became significant after day 50 (*p* = 0.002). Among these, patients in the “high SHR + high GluCV” subgroup had the highest 1-year mortality.

### 3.5 The Discrimination Ability of Each Prediction Model for Patients With NSTEMI and STEMI

For NSTEMI patients, the combined model outperformed SHR alone across all glucose metabolic subgroups [NGR: 0.667 (0.611–0.723) vs 0.489 (0.432–0.546); Pre-DM: 0.646 (0.590–0.703) vs 0.525 (0.461–0.589); DM: 0.615 (0.592–0.638) vs 0.506 (0.481–0.531)]. However, no significant advantage was observed compared with GluCV alone [NGR: 0.667 (0.611–0.723) vs 0.668 (0.613–0.724); Pre-DM: 0.646 (0.590–0.703) vs 0.647 (0.591–0.703); DM: 0.615 (0.592–0.638) vs 0.615 (0.593–0.638)] (**Supplementary Table 18**).

For STEMI patients, SHR alone outperformed the combined model in the NGR subgroup [0.568 (0.451–0.685) vs 0.564 (0.463–0.666)], while the combined model performed better than GluCV alone [0.564 (0.463–0.666) vs 0.562 (0.460–0.664)]. In the Pre-DM subgroup, the combined model showed superior performance compared with SHR alone and GluCV alone [0.638 (0.527–0.749) vs 0.637 (0.526–0.747) vs 0.620 (0.504–0.735)]. In the DM subgroup, GluCV alone exhibited the best performance [0.620 (0.570–0.669) vs 0.617 (0.568–0.667)], and the combined model showed stronger predictive power than SHR alone [0.617 (0.568–0.667) vs 0.432 (0.378–0.486)] (**Supplementary Tables 19,20**).

### 3.6 Machine Learning: SHR, GV and 1-year Mortality in Patients With NSTEMI

The Boruta algorithm was used to identify the final variables included in the ML models for each glucose subgroup of NSTEMI patients. The Boruta algorithm selected 13, 14, and 16 features as optimal predictors of mortality for the NGR, Pre-DM, and DM subgroups, respectively (**Supplementary Figs. 11–13**). Results of the Boruta algorithm for the overall NSTEMI patient population are shown in **Supplementary Fig. 14**.

**Fig. 5.**
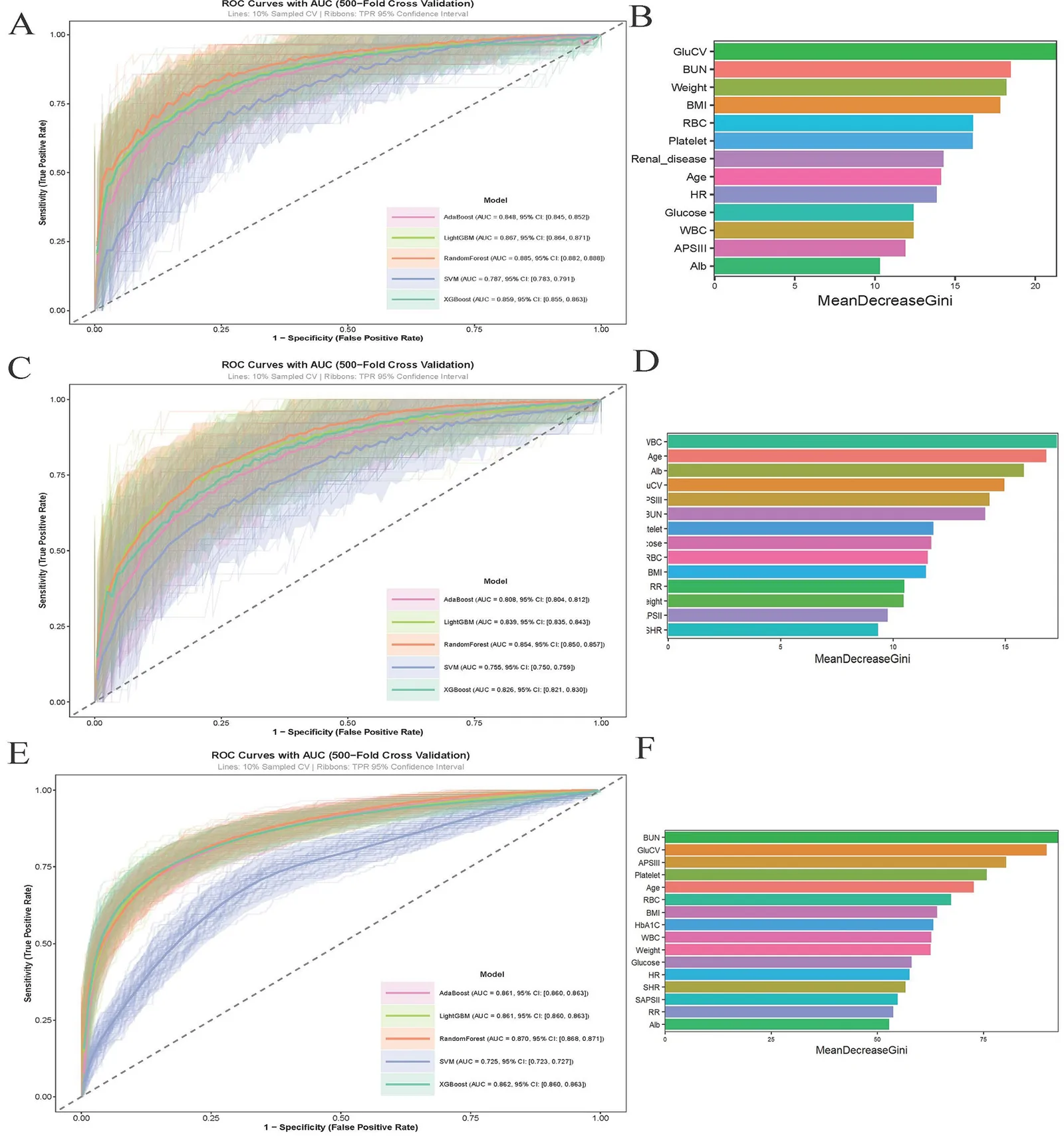
**Receiver operating characteristic (ROC) curves and importance ranking of machine learning (ML)-based 1-year mortality prediction models in NSTEMI patients**. (A,B) Patients with NGR; (C,D) Patients with Pre‑DM; (E,F) Patients with DM. SVM, support vector machine; Adaboost, adaptive boosting; RF, random forest; XGBoost, extreme gradient boosting; LightGBM, light gradient-boosted machine.

The RF model exhibited excellent performance in the NGR subgroup (AUC: 0.885, 95% CI: 0.882–0.888; Fig. 5A). Feature importance ranking for the RF model showed GluCV as the top feature, while SHR was not included in the model (Fig. 5B). The RF model also performed well in the Pre-DM subgroup (AUC: 0.854, 95% CI: 0.85–0.857; Fig. 5C), with GluCV ranked 4th and SHR ranked last in feature importance (Fig. 5D). In the DM subgroup, the RF model remained the best-performing model (AUC: 0.87, 95% CI: 0.868–0.871; Fig. 5E), with GluCV ranked 2nd and SHR ranked 4th from the bottom (Fig. 5F).

## 4. Discussion

The management of acute myocardial infarction necessitates precise risk stratification [11,12,13], yet the integration of dynamic glycemic measures with underlying metabolic phenotypes remains inadequately explored [14,15,16]. This study advances this field by conducting a rigorous glycometabolic phenotype-stratified analysis of SHR and GluCV in a large cohort of well-characterized NSTEMI and STEMI patients, uncovering context-dependent prognostic signals for these glycemic metrics and identifying a novel high GluCV + low SHR high-risk phenotype in NSTEMI.

A central finding of this work is the divergent prognostic utility of GluCV and SHR between NSTEMI and STEMI, with GluCV demonstrating more consistent associations with 1-year mortality across all NSTEMI glycometabolic subgroups and a significant non-linear dose-response relationship. In contrast, SHR was largely non-significant in fully adjusted NSTEMI analyses and exhibited subgroup-restricted prognostic value in STEMI (significant only in DM patients). While these results suggest GluCV may be a more informative glycemic metric for NSTEMI risk stratification, this conclusion requires extreme caution due to two key confounding factors that limit inference of true pathophysiological relevance. First, GluCV is calculated from serial glucose measurements, and its magnitude may reflect the intensity of glucose monitoring (a marker of clinical concern for severe illness) rather than inherent glycemic variability. More severely ill patients undergo more frequent testing, which can lead to higher GluCV values and potential overestimation of its prognostic signal. Second, GluCV is strongly correlated with acute illness severity scores. Although we adjusted for major severity-of-illness covariates, residual confounding by unmeasured disease severity may remains. It is therefore unclear whether GluCV is an independent prognostic marker or a surrogate for more severe myocardial and systemic injury in acute MI.

In patients with NSTEMI, the lack of a significant SHR and mortality in adjusted analyses likely reflects the limitations of SHR as a static measure: it captures only admission hyperglycemia relative to chronic glycemia and dose not account for the dynamic glycemic fluctuations that characterize the acute phase of NSTEMI, a subtype defined by persistent myocardial ischemia, subtotal coronary occlusions, and a chronic low-grade inflammatory milieu [17,18]. These pathophysiological features render dynamic glycemic variability (measured by GluCV) a more relevant marker of ongoing injury, whereas SHR fails to capture this temporal dimension. In STEMI, SHR’s significant association with mortality only in DM patients reflects the confluence of pre-existing vascular vulnerability, insulin resistance, and stress-induced hyperglycemia [19,20], which amplifies the toxic effects of acute hyperglycemia in this high-risk group. For non-diabetic STEMI patients, the dominant effect of time-to-reperfusion, the single most important determinant of STEMI outcomes [21] likely overshadows the modest prognostic signal of SHR, explaining its non-significance in the NGR/Pre-DM subgroups. Importantly, our results do not support SHR as a universal prognostic metric; its utility is limited to diabetic STEMI cohorts, and it should not be used as a standalone risk marker in other MI populations.

The muted response to high GluCV in Pre-DM patients with NSTEMI warrants particular attention. This finding may not reflect a benign response but instead suggests a state of “compensated dysglycemia”, in which preserved pancreatic beta-cell function pemits greater tolerance to glucose fluctuations without immediate translation into increased mortality risk within our 1-year follow-up period. This observation underscores the importance of glycemic phenotype in interpreting variability metrics [22,23].

Moving beyond individual metrics, our study is the first to report a synergistic risk phenotype—high GluCV + low SHR—across all NSTEMI glycometabolic subgroups and in NGR/DM STEMI patients. This counterintuitive “high‑variability, low‑stress” profile is only a phenomenological observation, not a confirmed mechanistic relationship, and is subject to the same confounding factors as the individual metrics (monitoring intensity, disease severity). We hypothesize this profile may signal a metabolically suppressed state where intense physiological stress (high GluCV, reflecting stress-induced hyperglycemia) coexists with a blunted metabolic response or aggressive insulin therapy that minimizes glucose fluctuations (low SHR) [24,25,26]. This combination could create a lethal mismatch between myocardial energy demand and supply—high SHR indicates increased metabolic demand, while low GluCV reflects a loss of metabolic flexibility—exacerbating myocardial ischemia in NSTEMI patients with pre-existing subtotal coronary occlusions. The time-varying hazard ratio in NSTEMI patients with DM, with significant mortality differences emerging only after day 200 post-discharge, further suggests this phenotype’s prognostic signal may be mediated by long-term adverse cardiac remodeling [27] rather than acute MI pathophysiology, an untested hypothesis that requires prospective validation. This high GluCV + low SHR phenotype is therefore a hypothesis-generating finding; future studies are needed to confirm its existence in independent cohorts, unravel disentangle its underlying mechanisms, and test its modifiability with targeted glycemic management.

To translate insights into clinical practice, we developed ML models for NSTEMI, with the Random Forest algorithm achieving the highest performance. Feature importance analysis confirmed GluCV as a top predictor, whereas SHR had less importance. These ML models serve as a proof-of-concept that integrating stratified glycemic metrics into MI risk prediction may improve accuracy, but they are not ready for clinical use—feature importance does not imply causality, and phenotype-specific feature selection requires prospective validation [28,29]. While our study emphasizes integrated SHR-GluCV assessment, Random Forest feature importance ranked GluCV as the top predictor, with SHR low-ranked or excluded from optimal feature sets in NSTEMI. This apparent contradiction reflects SHR’s phenotype-specific prognostic utility and the dimensional complementarity between SHR and GluCV, the core of integrated assessment’s superiority over GluCV-centric evaluation. SHR’s low global statistical weight is rational: it is not a generalized marker, with prognostic value restricted to diabetic STEMI patients in fully adjusted analyses. Critically, statistical weight does not equal clinical incremental value: SHR improves reclassification of intermediate-to-high risk NSTEMI patients and is indispensable for identifying the high GluCV + low SHR phenotype—a subgroup the GluCV-only model cannot define, as it cannot distinguish metabolic blunting from therapeutic glycemic control underlying low GluCV. The integrated approach outperforms single GluCV assessment by aligning with acute dysglycemia’s pathophysiology (GluCV: dynamic injury; SHR: static stress imbalance) and enabling phenotype-specific clinical management: e.g., high SHR + low GluCV patients require metabolic/energy balance evaluation, not just intensified variability control. This also validates our glycometabolic phenotype + MI subtype stratification—global ML models cannot capture SHR’s subtype-specific value, while integration unifies statistical prediction and clinical interpretability.

A key inherent limitation of this study is the significant sample size imbalance (NSTEMI: n = 3628; STEMI: n = 825), with STEMI’s NGR (n = 128) and Pre-DM (n = 117) subgroups markedly smaller than its DM subgroup (n = 580). NSTEMI patients have higher ICU admission rates/longer stays due to insidious onset and comorbidity burden, while STEMI patients receive urgent revascularization with stricter ICU criteria. Cross-subtype consistency of the SHR-GluCV phenotype is only validated in the DM subgroup, and fine stratified/sensitivity analyses (e.g., by insulin therapy) are unfeasible in small STEMI subgroups. To mitigate this, we prioritized 95% CI reporting for STEMI, avoided definitive “no association” conclusions, and restricted cross-subtype comparisons to the power-sufficient DM subgroup. Importantly, this limitation only affects STEMI analysis depth and does not undermine core study conclusions: GluCV as a core NSTEMI prognostic marker, the high GluCV + low SHR high‑risk NSTEMI phenotype, and the Random Forest model’s high NSTEMI predictive accuracy are all based on the large, power-sufficient NSTEMI cohort, ensuring stability and reliability.

### Limitation

Ultimately, this hypothesis-generating retrospective study demonstrates that SHR and GluCV’s prognostic utility in acute MI is highly dependent on infarction subtype and baseline glycemic phenotype. Our findings challenge universal glycemic metrics in MI, and while our observational design precludes immediate clinical recommendations, it underscores that precision medicine requires moving beyond unstratified analyses to account for the biological heterogeneity of MI and glycometabolic states.

## 5. Conclusion

This hypothesis-generating retrospective study demonstrates that the prognostic utility of SHR and GluCV in acute MI is highly dependent on infarction subtype (NSTEMI vs. STEMI) and baseline glycemic phenotype (NGR, Pre-DM, DM). Our key exploratory findings and resulting hypotheses include:

(1) GluCV: Showed consistent prognostic associations with 1-year mortality in NSTEMI across all glycemic subgroups. Future studies should test if mitigating GluCV improves NSTEMI outcomes, while carefully adjusting for monitoring intensity and disease severity. (2) SHR: Exhibited limited general utility but significant prognostic value specifically in diabetic STEMI patients, suggesting its selective inclusion in future risk tools. (3) Composite Metrics: Identified a novel, counterintuitive high-risk phenotype(High GluCV + Low SHR). Prospective research is needed to validate this and elucidate its mechanisms (e.g., metabolic blunting). (4) ML Models: Confirmed GluCV as a prominent predictor for NSTEMI, pending external validation for clinical use.

## Data Availability

Data for this study were obtained from the MIMIC database. Because all MIMIC data access must be secured via formal application, we cannot publicly share the specific data used in this study. However for qualified researchers who have successfully completed the application process for MIMIC database access, the corresponding author (Xiaoping Fan) is willing to provide the study-related data upon receiving a reasonable request.

## Abbreviations

NSTEMI, non-ST-segment elevation myocardial infarction; STEMI, ST-segment elevation myocardial infarction; GluCV, blood glucose coefficient of variation; SHR, stress hyperglycemia ratio; NGR, normal glucose regulation; Pre-DM, pre-diabetes; DM, diabetes mellitus; ICU, intensive care unit; MIMIC-IV, Medical Information Mart for Intensive Care IV; APSIII, Acute Physiology Score III; LODS, Logistic Organ Dysfunction System; OASIS, Oxford acute severity of illness score; SAPSII, Simplified Acute Physiology Score II; HR, heart rate; ML, machine learing.
