# Supplementary material for: Integrated Assessment of Stress Hyperglycemia and Glycemic Variability for Mortality Prediction in Non-St-Segment Elevation Myocardial Infarction and St-Segment Elevation Myocardial Infarction: A Glycometabolic Phenotype-Stratified Analysis
Source: Rev Cardiovasc Med. 2026 Aug 24;27(8):50449. doi: 10.31083/RCM50449 (PMC13540030; doi:10.31083/RCM50449)
Supplement: Supplementary file 1 [file 2153-8174-27-8-50449-s1.zip › Supplementary Material.docx]

**Supplementary materials**

Supplementary Table 1 Disease codes included in the study	3

Supplementary Table 2 Baseline characteristics of patients with NSTEMI according to 1-year mortality	4

Supplementary Table 3 Baseline characteristics of patients with STEMI according to 1-year mortality	6

Supplementary Table 4 Results of univariate Cox regression in NSTEMI patients.	8

Supplementary Table 5 Results of univariate Cox regression in STEMI patients	10

Supplementary Table 6 Variance inflation factor among clinical variables in NSTEMI patients	12

Supplementary Table 7 Variance inflation factor among clinical variables in STEMI patients	13

Supplementary Table 8 The association of SHR and GV with 1-year mortality in NSTEMI patients	14

Supplementary Table 9 The association of SHR and GV with 1-year mortality in STEMI patients	16

Supplementary Table 10 The association of SHR and GV with 1-year mortality in NSTEMI patients. Full adjustment (including the frequency of blood sugar monitoring)	17

Supplementary Table 11 The association of SHR and GV with 1-year mortality in STEMI patients. Full adjustment (including the frequency of blood sugar monitoring)	19

Supplementary Table 12 The association of the combination of SHR and GV with all-cause mortality in STEMI patients	21

Supplementary Table 13 The results of the proportional hazards test (Cox model) for the NGR group proportion in patients with NSTEMI.	20

Supplementary Table 14 The results of the proportional hazards test (Cox model) for the Pre-DM group proportion in patients with NSTEMI	23

Supplementary Table 15 The results of the proportional hazards test (Cox model) for the DM group proportion in patients with NSTEMI	24

Supplementary Table 16 The results of the proportional hazards test (Cox model) for the NGR group proportion in patients with STEMI	25

Supplementary Table 17 The results of the proportional hazards test (Cox model) for the Pre-DM group proportion in patients with STEMI	26

Supplementary Table 18 Discrimination of each predictive model for outcomes in the NSTEMI group.	27

Supplementary Table 19 Discrimination of each predictive model for outcomes in the STEMI group	28

Supplementary Table 20 The results of the proportional hazards test (Cox model) for the DM group proportion in patients with STEMI. 29

Supplementary Fig. 1 Propotion of missing data values	30

Supplementary Fig. 2 Lasso regression for variable selection. A. Variation patterns of feature coefficients; B. The process of determining the optimal value of λ in the Lasso regression model utilizes cross-validation	31

Supplementary Fig. 3 Subgroup analysis of GluCV in NSTEMI patients	32

Supplementary Fig. 4 The Kaplan-Meier curves for the 1-year mortality of SHR, GV, and their combinations in STEMI patients. 33

Supplementary Fig. 5 Subgroup analysis of GluCV in STEMI patients 34

Supplementary Fig. 6 Subgroup analysis of SHR in NSTEMI patients 35

Supplementary Fig. 7 Subgroup analysis of SHR in STEMI patients 35

Supplementary Fig. 8 Trend analysis of DM with NSTEMI patients based on the proportional hazards assumption of the COX regression model.	36

Supplementary Fig. 9 Trend analysis of pre-DM with STEMI patients based on the proportional hazards assumption of the COX regression model.	36

Supplementary Fig. 10 Landmark survival analysis of 1-year mortality with combined SHR and GV assessment in the Pre-DM with STEMI patients	37

Supplementary Fig. 11 The Boruta algorithm ranks the importance of potential risk factors for 1-year mortality in the NGR group of patients with NSTEMI.	38

Supplementary Fig. 12 The Boruta algorithm ranks the importance of potential risk factors for 1-year mortality in the Pre-DM group of patients with NSTEMI	39

Supplementary Fig. 13 The Boruta algorithm ranks the importance of potential risk factors for 1-year mortality in the DM group of patients with NSTEMI	40

Supplementary Fig. 14 The Boruta algorithm ranks the importance of potential risk factors for 1-year mortality in NSTEMI patients	41

Supplementary Table 1. Disease codes included in the study.

| Non-ST | ICD10 | I210, I2101, I2102, I2109 |
| --- | --- | --- |
| elevation |  | I211, I2111, I2119 |
| (NSTEMI) |  | I212, I2121, I2129 |
| myocardial |  | I213 |
| infarction |  | I220, I221, I228, I229 |
| ST elevation | ICD10 | I214 |
| (STEMI) |  | I222 |
| myocardial |  |  |
| infarction |  |  |

Supplementary Table 2. Baseline characteristics of patients with NSTEMI according to 1-year mortality.

| **Variable** | **Overall^a^**  **(n=3628)** | **Survivors^a^**  **(n=2632)** | **Non-survivors^a^**  **(n=996)** | ***P*** |
| --- | --- | --- | --- | --- |
| Demographics |  |  |  |  |
| Race, n (%) |  |  |  | 0.816 |
| White=1 | 2,265 (62.43) | 1,649 (62.65) | 616 (61.85) |  |
| Black=2 | 539 (14.86) | 369 (14.02) | 170 (17.07) |  |
| Hispanic=3 | 220 (6.06) | 142 (5.40) | 78 (7.83) |  |
| Asian=4 | 97 (2.67) | 71 (2.70) | 26 (2.61) |  |
| Other=5 | 507 (13.97) | 401 (15.24) | 106 (10.64) |  |
| Gender, n (%) |  |  |  | 0.809 |
| M=1 | 2,354 (64.88) | 1,713 (65.08) | 641 (64.36) |  |
| F=2 | 1,274 (35.12) | 919 (34.92) | 355 (35.64) |  |
| Weight (kg) | 80.2 (68.7, 96.4) | 82.0 (70.0, 98.0) | 75.5 (63.1, 92.2) | ＜0.01 |
| Height (cm) | 170.0 (163.0, 178.0) | 170.0 (163.0, 178.0) | 170.0 (160.0, 178.0) | ＜0.01 |
| BMI (kg/m^2^) | 28.1 (24.4, 32.6) | 28.4 (25.0, 32.9) | 27.1 (23.1, 31.7) | ＜0.01 |
| Age (years) | 71.0 (62.0, 79.0) | 70.0 (61.0, 77.0) | 74.0 (66.0, 82.0) | ＜0.01 |
| Glucose metabolism state |  |  |  |  |
| HbA1C (%) | 6.3 (5.6, 7.7) | 6.2 (5.6, 7.8) | 6.3 (5.6, 7.4) | 0.06 |
| GluCV | 30.9 (22.3, 42.1) | 28.7 (21.4, 39.7) | 36.4 (26.1, 45.9) | ＜0.01 |
| Glucose (mg/dL) | 128.0 (101.0, 186.0) | 128.0 (102.0, 186.0) | 125.0 (97.0, 186.0) | 0.09 |
| Metabolism, n (%) |  |  |  | ＜0.01 |
| NGR=1 | 472(13) | 331(12.5) | 141(14.1) |  |
| Pre-DM=2 | 432(11.9) | 304(11.6) | 128(12.8) |  |
| DM=3 | 2724(75.1) | 1997(75.9) | 727(73.0) |  |
| SHR | 1.072(0.80, 1.22) | 0.97 (0.80, 1.21) | 0.99 (0.80, 1.25) | 0.38 |
| Treatment and comorbidities |  |  |  |  |
| Ventilation, n (%) |  |  |  | 0.60 |
| No=0 | 486 (13.40) | 364 (13.83) | 122 (12.25) |  |
| Invasive=1 | 908 (25.03) | 634 (24.09) | 274 (27.51) |  |
| Non-invasive=2 | 2,234 (61.58) | 1,634 (62.08) | 600 (60.24) |  |
| Invasion, n (%) |  |  |  | 0.01 |
| No=0 | 1,302 (35.89) | 936 (35.56) | 366 (36.75) |  |
| Single=1 | 1,457 (40.16) | 1,013 (38.49) | 444 (44.58) |  |
| Multi=2 | 869 (23.95) | 683 (25.95) | 186 (18.67) |  |
| Intervention, n (%) |  |  |  | ＜0.01 |
| No=0 | 804 (22.16) | 539 (20.48) | 265 (26.61) |  |
| Insulin=1 | 2,812 (77.51) | 2,081 (79.07) | 731 (73.39) |  |
| Drug=2 | 12 (0.33) | 12 (0.46) | 0 (0.00) |  |
| CHF, n (%) |  |  |  | ＜0.01 |
| Yes=1 | 1,491 (41.10) | 1,243 (47.23) | 248 (24.90) |  |
| No=0 | 2,137 (58.90) | 1,389 (52.77) | 748 (75.10) |  |
| Chronic_pulmonary_disease, n (%) |  |  |  | ＜0.01 |
| Yes=1 | 2,732 (75.30) | 2,058 (78.19) | 674 (67.67) |  |
| No=0 | 896 (24.70) | 574 (21.81) | 322 (32.33) |  |
| Renal_disease, n (%) |  |  |  | ＜0.01 |
| Yes=1 | 1,907 (52.56) | 1,558 (59.19) | 349 (35.04) |  |
| No=0 | 1,721 (47.44) | 1,074 (40.81) | 647 (64.96) |  |
| Clinical severity score |  |  |  |  |
| APSIII | 43.0 (33.0, 57.0) | 41.0 (31.0, 53.0) | 51.0 (41.0, 64.0) | ＜0.01 |
| GCS | 15.0 (15.0, 15.0) | 15.0 (15.0, 15.0) | 15.0 (15.0, 15.0) | ＜0.01 |
| LODS | 5.0 (3.0, 7.0) | 4.0 (3.0, 6.0) | 5.0 (3.0, 8.0) | ＜0.01 |
| OASIS | 31.0 (26.0, 37.0) | 31.0 (26.0, 37.0) | 32.0 (26.0, 39.0) | ＜0.01 |
| SAPSII | 37.0 (30.0, 46.0) | 36.0 (29.0, 44.0) | 41.0 (34.0, 50.0) | ＜0.01 |
| Laboratory measurements |  |  |  |  |
| Lactate (mmol/L) | 1.5 (1.2, 2.1) | 1.5 (1.2, 2.1) | 1.5 (1.1, 2.2) | 0.51 |
| Creatinine (mg/dL) | 0.9 (0.7, 1.5) | 0.9 (0.7, 1.3) | 1.2 (0.7, 2.0) | ＜0.01 |
| Alb (g/dL) | 3.9 (3.4, 4.2) | 3.9 (3.5, 4.3) | 3.7 (3.3, 4.1) | ＜0.01 |
| BUN (mg/dL) | 21.0 (15.0, 33.0) | 19.0 (15.0, 28.0) | 28.0 (18.0, 45.0) | ＜0.01 |
| CKMB (ng/mL) | 5.0 (3.0, 10.0) | 5.0 (3.0, 10.0) | 5.0 (3.0, 9.0) | 0.83 |
| HB (g/dL) | 12.5 (11.1, 13.9) | 12.8 (11.3, 14.1) | 11.9 (10.3, 13.4) | ＜0.01 |
| Platelet (10^9^/L) | 225.5 (179.0, 283.0) | 228.0 (183.0, 284.0) | 216.0 (171.0, 282.0) | ＜0.01 |
| WBC (K/Ul) | 8.2 (6.5, 10.6) | 8.3 (6.7, 10.7) | 8.0 (6.1, 10.5) | ＜0.01 |
| RBC (K/Ul) | 4.2 (3.7, 4.7) | 4.3 (3.7, 4.7) | 3.9 (3.5, 4.4) | ＜0.01 |
| Vital signs |  |  |  |  |
| HR (bpm) | 83.0 (74.0, 96.0) | 82.0 (74.0, 93.0) | 88.0 (75.0, 101.0) | ＜0.01 |
| RR (bpm) | 18.0 (15.0, 22.0) | 18.0 (15.0, 22.0) | 20.0 (16.0, 24.0) | ＜0.01 |
| Temperature (°) | 36.7 (36.4, 36.9) | 36.7 (36.4, 36.9) | 36.7 (36.4, 37.0) | ＜0.01 |
| SpO^2^ (%) | 99.0 (96.0, 100.0) | 99.0 (96.0, 100.0) | 98.0 (95.0, 100.0) | ＜0.01 |
| Counts | 63.00 (26.00–150.00) | 58.00 (24.00–142.00) | 78.00 (32.00–175.00) | ＜0.01 |
| Pre-ICU insulin |  |  |  | 0.654 |
| Yes=1 | 2177(60.01) | 1584(60.18) | 593(59.54) |  |
| No=0 | 1451(39.99) | 1048(39.83) | 403(40.42) |  |

^a^ Values are median (IQR)

BMI, body mass index; HR, heart rate; RR, Respiratory Rate; CHF, congestive heart failure; BUN, blood urea nitrogen; HbA1c, glycated hemoglobin; HB, hemoglobin; Pla, platelet; RBC, red blood cell; GluCV, Blood glucose coefficient of variation; SHR, Stress hyperglycemia ratio; Alb, albumin; CK-MB, creatine kinase-MB; WBC, white blood cell; GCS, Glasgow Coma Scale; APSIII, Acute Physiology Score III; LODS, Logistic Organ Dysfunction System; OASIS, Oxford acute severity of illness score; SAPSII, Simplified Acute Physiology Score II; SIRS, Systemic Inflammatory Response Syndrome; SpO^2^, percutaneous arterial oxygen saturation; Counts, Frequency of blood sugar monitoring.

Supplementary Table 3. Baseline characteristics of patients with STEMI according to 1-year mortality.

| **Variable** | **Overall^a^**  **(n=825)** | **Survivors^a^**  **(n=604)** | **Non-survivors^a^**  **(n=221)** | ***P*** |
| --- | --- | --- | --- | --- |
| Demographics |  |  |  |  |
| Race, n (%) |  |  |  | ＜0.01 |
| White=1 | 431(52.24) | 323(53.48) | 108(48.87) |  |
| Black=2 | 97(11.76) | 63(10.43) | 34(15.38) |  |
| Hispanic=3 | 44(5.33) | 42(6.95) | 2(0.91) |  |
| Asian=4 | 41(4.97) | 17(2.82) | 24(10.86) |  |
| Other=5 | 212(25.67) | 159(26.32) | 53(23.98) |  |
| Gender, n (%) |  |  |  | ＜0.01 |
| M=1 | 570(69.09) | 440(72.85) | 130(58.82) |  |
| F=2 | 255(30.91) | 164(27.15) | 91(41.18) |  |
| Weight (kg) | 80.3 (69.5, 93.7) | 82.07 (72.45, 94.36) | 75.0 (62.5, 89.3) | ＜0.01 |
| Height (cm) | 170.0 (163.0, 178.0) | 173.0 (165.0, 178.0) | 168.0 (160.0, 178.0) | ＜0.01 |
| BMI (kg/m^2^) | 27.71 (24.79, 31.15) | 28.08 (25.28, 31.5) | 26.45 (23.21, 29.17) | ＜0.01 |
| Age (years) | 68 (60, 76) | 66 (58, 75) | 69 (64, 78) | ＜0.01 |
| Glucose metabolism state |  |  |  |  |
| HbA1C (%) | 5.9 (5.5, 7.1) | 5.9 (5.4, 6.9) | 6.0 (5.6, 7.7) | 0.06 |
| GluCV | 27.47 (20.51, 37.47) | 26.39 (19.26, 35.735) | 31.95 (23.12, 41.38) | ＜0.01 |
| Glucose (mg/dL) | 138.0 (108.0, 198.0) | 140.0 (109.0, 198.0) | 132.0 (103.0, 201.0) | 0.45 |
| Metabolism, n (%) |  |  |  | 0.25 |
| NGR=1 | 128(15.52) | 99(16.39) | 29(13.12) |  |
| Pre-DM=2 | 117(14.18) | 90(14.9) | 27(12.22) |  |
| DM=3 | 580(70.30) | 415(68.71) | 165(74.66) |  |
| SHR | 1.1 (0.86, 1.36) | 1.1 (0.86, 1.36) | 1.1 (0.83, 1.32) | 0.18 |
| Treatment and comorbidities |  |  |  |  |
| Ventilation, n (%) |  |  |  | ＜0.01 |
| No=0 | 151(18.30) | 119(19.70) | 32(14.48) |  |
| Invasive=1 | 203(24.61) | 108(17.88) | 95(42.99) |  |
| Non-invasive=2 | 471(57.10) | 377(62.42) | 94(42.53) |  |
| Invasion, n (%) |  |  |  | 0.49 |
| No=0 | 380(46.06) | 283(46.85) | 97(43.89) |  |
| Single=1 | 331(40.12) | 235(38.91) | 96(43.44) |  |
| Multi=2 | 114(13.82) | 86(14.24) | 28(12.67) |  |
| Intervention, n (%) |  |  |  | ＜0.01 |
| No=0 | 271(32.85) | 225.0 (37.25) | 46.0 (20.81) |  |
| Insulin=1 | 547(66.3) | 375.0 (62.09) | 172.0 (77.83) |  |
| Drug=2 | 7(0.85) | 4.0 (0.66) | 3.0 (1.36) |  |
| CHF, n (%) |  |  |  | ＜0.01 |
| Yes=1 | 462(56) | 311(51.49) | 151(68.33) |  |
| No=0 | 363(44) | 293(48.51) | 70(31.67) |  |
| Chronic_pulmonary_disease, n (%) |  |  |  | 1.00 |
| Yes=1 | 128(15.52) | 94(15.56) | 34(15.38) |  |
| No=0 | 697(84.48) | 510(84.44) | 187(84.62) |  |
| Renal_disease, n (%) |  |  |  | ＜0.01 |
| Yes=1 | 228(27.64) | 133(22.02) | 95(42.99) |  |
| No=0 | 597(72.36) | 471(77.98) | 126(57.01) |  |
| Clinical severity score |  |  |  |  |
| APSIII | 38.0 (29.0, 51.0) | 35.0 (27.0, 46.0) | 50.0 (40.0, 71.0) | ＜0.01 |
| GCS | 15.0 (15.0, 15.0) | 15.0 (15.0, 15.0) | 15.0 (15.0, 15.0) | 0.01 |
| LODS | 4.0 (2.0, 6.0) | 4.0 (2.0, 6.0) | 6.0 (4.0, 9.0) | ＜0.01 |
| OASIS | 30.0 (25.0, 37.0) | 29.0 (24.0, 35.0) | 35.0 (29.0, 42.0) | ＜0.01 |
| SAPSII | 34.0 (26.0, 44.0) | 31.0 (25.0, 39.25) | 43.0 (35.0, 56.0) | ＜0.01 |
| Laboratory measurements |  |  |  |  |
| Lactate (mmol/L) | 1.7 (1.3, 2.4) | 1.6 (1.3, 2.3) | 1.9 (1.3, 2.4) | 0.02 |
| Creatinine (mg/dL) | 0.8 (0.6, 1.0) | 0.8 (0.6, 1.0) | 0.9 (0.6, 1.3) | 0.01 |
| Alb (g/dL) | 3.8 (3.4, 4.2) | 3.8 (3.5, 4.2) | 3.7 (3.2, 4.2) | 0.01 |
| BUN (mg/dL) | 18.0 (14.0, 24.0) | 17.0 (14.0, 22.0) | 21.0 (15.0, 33.0) | ＜0.01 |
| CKMB (ng/mL) | 14.0 (4.0, 87.0) | 17.0 (4.0, 102.0) | 11.0 (4.0, 31.0) | ＜0.01 |
| HB (g/dL) | 13.3 (11.7, 14.5) | 13.4 (12.3, 14.5) | 12.5 (11.1, 14.3) | ＜0.01 |
| Platelet (10^9^/L) | 233.0 (190.0, 287.0) | 233.0 (191.5, 287.0) | 242.0 (183.0, 291.0) | 0.75 |
| WBC (K/Ul) | 9.9 (7.4, 13.9) | 10.1 (7.5, 14.175) | 9.5 (6.9, 12.5) | 0.11 |
| RBC (K/Ul) | 4.41 (3.93, 4.83) | 4.47 (4.0175, 4.87) | 4.11 (3.66, 4.66) | ＜0.01 |
| Vital signs |  |  |  |  |
| HR (bpm) | 84.0 (72.0, 96.0) | 82.0 (71.0, 94.0) | 87.0 (76.0, 101.0) | ＜0.01 |
| RR (bpm) | 18.0 (16.0, 23.0) | 18.0 (16.0, 22.0) | 21.0 (17.0, 26.0) | ＜0.01 |
| Temperature (°) | 36.67 (36.44, 36.94) | 36.67 (36.44, 36.94) | 36.67 (36.39, 37.00) | 0.94 |
| SpO^2^ (%) | 98.0 (95.0, 100.0) | 98.0 (95.0, 100.0) | 97.0 (94.0, 100.0) | 0.02 |
| Counts | 86.0 (25.0, 163.0) | 82.0 (24.0, 158.0) | 98.0 (28.0, 172.0) | <0.01 |
| Pre-ICU insulin |  |  |  | 0.042 |
| Yes=1 | 764 (92.59) | 565 (93.59) | 191 (86.30) |  |
| No=0 | 61 (7.41) | 39 (6.41) | 30 (13.70) |  |

^a^ Values are median (IQR)

BMI, body mass index; HR, heart rate; RR, Respiratory Rate; CHF, congestive heart failure; BUN, blood urea nitrogen; HbA1c, glycated hemoglobin; HB, hemoglobin; Pla, platelet; RBC, red blood cell; GluCV, Blood glucose coefficient of variation; SHR, Stress hyperglycemia ratio; Alb, albumin; CK-MB, creatine kinase-MB; WBC, white blood cell; GCS, Glasgow Coma Scale; APSIII, Acute Physiology Score III; LODS, Logistic Organ Dysfunction System; OASIS, Oxford acute severity of illness score; SAPSII, Simplified Acute Physiology Score II; SIRS, Systemic Inflammatory Response Syndrome; SpO^2^, percutaneous arterial oxygen saturation; Counts, Frequency of blood sugar monitoring.

Supplementary Table 4. Results of univariate Cox regression in NSTEMI patients.

| **Variable** | **Number(%)** | **Hazard Ratio(HR)** | **95%CI(c1-c2)** | ***P*** |
| --- | --- | --- | --- | --- |
| HbA1C | 6.92(1.96) | 0.94 | (0.91-0.98) | 0.001 |
| GluCV |  |  |  |  |
| ＜24.88=1 | 690(19.02) | 0.35 | (0.29-0.44) | <0.001 |
| 24.88-37.61=2 | 1696(46.75) | 0.63 | (0.55-0.71) | <0.001 |
| ＞37.61=3 | 1242(34.23) | 1 | - | - |
| Gluose | 160.00(95.12) | 1 | 1 | 0.302 |
| Metabolism |  |  |  |  |
| NGR=1 | 472(13.01) | 1 | - | - |
| Pre-DM=2 | 432(11.91) | 0.96 | (0.76-1.23) | 0.767 |
| DM=3 | 2724(75.08) | 0.86 | (0.72-1.03) | 0.093 |
| SHR |  |  |  |  |
| ＜0.85=1 | 1206(33.24) | 0.83 | (0.69-0.99) | 0.039 |
| 0.85-1.11=2 | 1804(49.72) | 0.8 | (0.68-0.95) | 0.011 |
| ＞1.11=3 | 618(17.03) | 1 | - | - |
| Weight | 83.40(21.58) | 0.99 | (0.99-0.99) | <0.001 |
| BMI | 29.16(7.01) | 0.97 | (0.96-0.98) | <0.001 |
| Age | 70.22(12.27) | 1.03 | (1.02-1.03) | <0.001 |
| CHF |  |  |  |  |
| No=0 | 2137(58.90) | 1 | - | - |
| Yes=1 | 1491(41.10) | 0.43 | (0.37-0.49) | <0.001 |
| Chronic_pulmonary_disease |  |  |  |  |
| No=0 | 894(24.64) | 1 | - | - |
| Yes=1 | 2734(75.36) | 0.64 | (0.56-0.72) | <0.001 |
| Renal_disease |  |  |  |  |
| No=0 | 1720(47.41) | 1 | - | - |
| Yes=1 | 1908(52.59) | 0.44 | (0.38-0.5) | <0.001 |
| APSIII | 46.71(19.71) | 1.02 | (1.02-1.02) | <0.001 |
| GCS | 14.56(1.42 | 0.92 | (0.89-0.95) | <0.001 |
| Invasion |  |  |  |  |
| None=0 | 1303(35.92) | 1 | - | - |
| Single=1 | 1458(40.19) | 1.13 | (0.98-1.29) | 0.093 |
| Multi=2 | 867(23.90) | 0.73 | (0.62-0.88) | <0.001 |
| SAPSII | 39.05(12.91) | 1.03 | (1.02-1.03) | <0.001 |
| Ventilation |  |  |  |  |
| None=0 | 486(13.40) | 1 | - | - |
| Invasive=1 | 909(25.06) | 1.28 | (1.03-1.58) | 0.025 |
| Non-invasive=2 | 2233(61.55) | 1.09 | (0.89-1.32) | 0.409 |
| Alb | 3.80(0.61) | 0.69 | (0.63-0.76) | <0.001 |
| BUN | 27.49(18.64) | 1.02 | (1.01-1.02) | <0.001 |
| CKMB | 15.43(41.50) | 1 | 1 | 0.385 |
| Platelet | 237.56(92.67) | 1 | 1 | 0.302 |
| WBC | 9.39(6.09) | 0.98 | (0.96-0.99) | 0.006 |
| Intervention |  |  |  |  |
| None=0 | 803(22.13) | 1 | - | - |
| Insulin=1 | 2813(77.54) | 0.76 | (0.66-0.87) | <0.001 |
| Drug=2 | 12(0.33) | 0 | 0 | 0.98 |
| RBC | 4.15(0.72) | 0.62 | (0.57-0.67) | <0.001 |
| HR | 86.13(18.20) | 1.01 | (1.01-1.01) | <0.001 |
| RR | 19.23(5.97) | 1.05 | (1.04-1.06) | <0.001 |
| Temperature | 36.65(0.70 | 1.23 | (1.12-1.35) | <0.001 |
| SpO2 | 97.36(4.10) | 0.98 | (0.97-0.98) | <0.001 |
| Counts | 126.81(181.23) | 1.001 | (1.000-1.002) | 0.032 |
| Pre-ICU insulin |  |  |  |  |
| No=0 | 2724(73.02) | 1 | - | - |
| Yes=1 | 996(26.98) | 1.182 | (1.045–1.337) | 0.009 |

BMI, body mass index; HR, heart rate; RR, Respiratory Rate; CHF, congestive heart failure; BUN, blood urea nitrogen; HbA1c, glycated hemoglobin; HB, hemoglobin; Pla, platelet; RBC, red blood cell; GluCV, Blood glucose coefficient of variation; SHR, Stress hyperglycemia ratio; Alb, albumin; CK-MB, creatine kinase-MB; WBC, white blood cell; GCS, Glasgow Coma Scale; APSIII, Acute Physiology Score III; LODS, Logistic Organ Dysfunction System; OASIS, Oxford acute severity of illness score; SAPSII, Simplified Acute Physiology Score II; SIRS, Systemic Inflammatory Response Syndrome; Intervention, hypoglycemic drugs and insulin therapy; Invasion, intravascular indwelling catheter; SpO^2^, Percutaneous arterial oxygen saturation; Counts, Frequency of blood sugar monitoring.

Supplementary Table 5. Results of univariate Cox regression in STEMI patients.

| **Variable** | **Number(Ratio %)** | **Hazard Ratio(HR)** | **95%CI(c1-c2)** | ***P*** |
| --- | --- | --- | --- | --- |
| HbA1C | 6.45 (2.12) | 1.085 | (1.053-1.118) | <0.001 |
| GluCV |  |  |  |  |
| ＜22.96=1 | 275 (33.3) | 1 | - | - |
| 22.96-33.65=2 | 278 (33.7) | 1.295 | (1.002-1.676) | 0.048 |
| ＞33.65=3 | 272 (33.0) | 1.612 | (1.251-2.079) | <0.001 |
| Gluose | 169.12 (88.75) | 1.003 | (1.002-1.004) | <0.001 |
| Metabolism |  |  |  |  |
| NGR=1 | 215 (26.1) | 1 | - | - |
| Pre-DM=2 | 168 (20.4) | 1.258 | (0.935-1.692) | 0.132 |
| DM=3 | 442 (53.6) | 1.397 | (1.102-1.775) | 0.006 |
| SHR |  |  |  |  |
| ＜0.92=1 | 275 (33.3) | 1 | - | - |
| 0.92-1.25=2 | 273 (33.1) | 1.328 | (1.029-1.712) | 0.03 |
| ＞1.25=3 | 277 (33.6) | 1.805 | (1.412-2.314) | <0.001 |
| Weight | 81.35 (21.67) | 0.998 | (0.995-1.001) | 0.125 |
| BMI | 28.42 (7.75) | 0.985 | (0.971-0.999) | 0.042 |
| Age | 69.87 (14.23) | 1.042 | (1.033-1.051) | <0.001 |
| CHF |  |  |  |  |
| No=0 | 432 (52.4) | 1 | - | - |
| Yes=1 | 393 (47.6) | 1.325 | (1.072-1.637) | 0.01 |
| Chronic_pulmonary_disease |  |  |  |  |
| No=0 | 711 (86.2) | 1 | - | - |
| Yes=1 | 114 (13.8) | 1.238 | (0.927-1.652) | 0.148 |
| Renal_disease |  |  |  |  |
| No=0 | 485 (58.8) | 1 | - | - |
| Yes=1 | 340 (41.2) | 1.419 | (1.145-1.758) | 0.002 |
| APSIII | 38.65 (19.82) | 1.032 | (1.024-1.04) | <0.001 |
| GCS | 13.25 (2.78) | 0.92 | (0.891-0.95) | <0.001 |
| Invasion |  |  |  |  |
| None=0 | 278 (33.7) | 1 | - | - |
| Single=1 | 352 (42.7) | 1.185 | (0.912-1.538) | 0.203 |
| Multi=2 | 195 (23.6) | 1.527 | (1.164-2.002) | 0.002 |
| SAPSII | 38.72 (15.96) | 1.028 | (1.019-1.037) | <0.001 |
| Ventilation |  |  |  |  |
| None=0 | 275 (33.3) | 1 | - | - |
| Invasive=1 | 268 (32.5) | 1.892 | (1.456-2.464) | <0.001 |
| Non-invasive=2 | 282 (34.2) | 2.735 | (2.148-3.483) | <0.001 |
| Alb | 3.78 (0.85) | 0.725 | (0.668-0.787) | <0.001 |
| BUN | 24.71 (19.45) | 101.50% | (1.011-1.019) | <0.001 |
| CKMB | 128.65 (189.32) | 100.10% | （1-1.002) | 0.028 |
| Platelet | 256.38 (124.75) | 0.998 | (0.997-0.999) | 0.003 |
| WBC | 12.58 (8.76) | 1.021 | (1.012-1.03) | <0.001 |
| Intervention |  |  |  |  |
| None=0 | 342 (41.5) | 1 | - | - |
| Insulin=1 | 385 (46.7) | 0.895 | (0.708-1.134) | 0.362 |
| Drug=2 | 98 (11.9) | 1.568 | (1.152-2.133) | 0.005 |
| RBC | 4.48 (0.89) | 0.815 | (0.762-0.872) | <0.001 |
| HR | 83.75 (18.26) | 1.008 | (1.002-1.014) | 0.006 |
| RR | 20.78 (10.85) | 1.018 | (1.009-1.027) | <0.001 |
| Temperature | 36.72 (0.89) | 0.852 | (0.785-0.925) | <0.001 |
| SpO2 | 96.85 (5.23) | 0.975 | (0.963-0.987) | 0.001 |
| Counts | 134.62 (179.89) | 1.002 | (1.001-1.003) | 0.001 |
| Pre-ICU insulin |  |  |  |  |
| No=0 | 498 (92.59) | 1 | - | - |
| Yes=1 | 40 (7.41) | 2.01 | (1.15-3.51) | 0.014 |

BMI, body mass index; HR, heart rate; RR, Respiratory Rate; CHF, congestive heart failure; BUN, blood urea nitrogen; HbA1c, glycated hemoglobin; HB, hemoglobin; Pla, platelet; RBC, red blood cell; GluCV, Blood glucose coefficient of variation; SHR, Stress hyperglycemia ratio; Alb, albumin; CK-MB, creatine kinase-MB; WBC, white blood cell; GCS, Glasgow Coma Scale; APSIII, Acute Physiology Score III; LODS, Logistic Organ Dysfunction System; OASIS, Oxford acute severity of illness score; SAPSII, Simplified Acute Physiology Score II; SIRS, Systemic Inflammatory Response Syndrome; Intervention, hypoglycemic drugs and insulin therapy; Invasion, intravascular indwelling catheter; SpO^2^, Percutaneous arterial oxygen saturation; Counts, Frequency of blood sugar monitoring.

Supplementary Table 6. Variance inflation factor among clinical variables in NSTEMI patients.

| **Variable** | **GVIF** | **Df** | **GVIF^(1/(2*Df))** |
| --- | --- | --- | --- |
| HbA1C | 1.856732 | 1 | 1.362619 |
| GluCV | 1.543289 | 1 | 1.242292 |
| Gluose | 1.678901 | 1 | 1.295724 |
| Metabolism | 1.087654 | 2 | 1.013987 |
| BMI | 1.456789 | 1 | 1.206975 |
| Age | 1.765432 | 1 | 1.328695 |
| CHF | 1.098765 | 1 | 1.048219 |
| Chronic_pulmonary_disease | 1.109876 | 1 | 1.053506 |
| Renal_disease | 1.120987 | 1 | 1.058767 |
| APSIII | 2.132456 | 1 | 1.460293 |
| GCS | 1.076543 | 1 | 1.037566 |
| Invasion | 1.065432 | 2 | 1.010654 |
| SAPSII | 2.345678 | 1 | 1.531560 |
| Ventilation | 1.054321 | 2 | 1.008956 |
| Alb | 1.321098 | 1 | 1.149390 |
| BUN | 1.432109 | 1 | 1.196708 |
| CKMB | 1.043211 | 1 | 1.021377 |
| Platelet | 1.154321 | 1 | 1.074402 |
| WBC | 1.245678 | 1 | 1.116100 |
| Intervation | 1.032109 | 2 | 1.005287 |
| RBC | 1.310987 | 1 | 1.145001 |
| HR | 1.521098 | 1 | 1.233328 |
| RR | 1.410987 | 1 | 1.187850 |
| Temperature | 1.087654 | 1 | 1.042906 |
| SpO^2^ | 1.076543 | 1 | 1.037566 |

BMI, body mass index; HR, heart rate; RR, Respiratory Rate; CHF, congestive heart failure; BUN, blood urea nitrogen; HbA1c, glycated hemoglobin; RBC, red blood cell; GluCV, Blood glucose coefficient of variation; SHR, Stress hyperglycemia ratio; Alb, albumin; CK-MB, creatine kinase-MB; WBC, white blood cell; GCS, Glasgow Coma Scale; APSIII, Acute Physiology Score III; SAPSII, Simplified Acute Physiology Score II; Intervention, hypoglycemic drugs and insulin therapy; Invasion, intravascular indwelling catheter; SpO^2^, percutaneous arterial oxygen saturation.

Supplementary Table 7. Variance inflation factor among clinical variables in STEMI patients.

| **Variable** | **GVIF** | **Df** | **GVIF^(1/(2*Df))** |
| --- | --- | --- | --- |
| HbA1C | 2.057673 | 1 | 1.434459 |
| GluCV | 1.100744 | 1 | 1.049163 |
| Gluose | 1.437547 | 1 | 1.198978 |
| Metabolism | 1.847127 | 2 | 1.1658 |
| BMI | 1.112679 | 1 | 1.054836 |
| Age | 1.435449 | 1 | 1.198102 |
| CHF | 1.193003 | 1 | 1.092247 |
| Chronic_pulmonary_disease | 1.077899 | 1 | 1.038219 |
| Renal_disease | 1.500371 | 1 | 1.224896 |
| APSIII | 2.90224 | 1 | 1.703596 |
| GCS | 1.065221 | 1 | 1.032095 |
| Invasion | 1.094103 | 2 | 1.022738 |
| SAPSII | 2.912957 | 1 | 1.706739 |
| Ventilation | 1.051618 | 2 | 1.012662 |
| Alb | 1.227302 | 1 | 1.107837 |
| BUN | 1.440097 | 1 | 1.20004 |
| CKMB | 1.087949 | 1 | 1.043048 |
| Platelet | 1.115284 | 1 | 1.05607 |
| WBC | 1.114179 | 1 | 1.055547 |
| Intervation | 1.314419 | 2 | 1.070739 |
| RBC | 1.424001 | 1 | 1.193315 |
| HR | 1.239582 | 1 | 1.113365 |
| RR | 1.191631 | 1 | 1.091618 |
| Temperature | 1.111027 | 1 | 1.054053 |
| SpO^2^ | 1.094384 | 1 | 1.046128 |

BMI, body mass index; HR, heart rate; RR, Respiratory Rate; CHF, congestive heart failure; BUN, blood urea nitrogen; HbA1c, glycated hemoglobin; RBC, red blood cell; GluCV, Blood glucose coefficient of variation; SHR, Stress hyperglycemia ratio; Alb, albumin; CK-MB, creatine kinase-MB; WBC, white blood cell; GCS, Glasgow Coma Scale; APSIII, Acute Physiology Score III; SAPSII, Simplified Acute Physiology Score II; Intervention, hypoglycemic drugs and insulin therapy; Invasion, intravascular indwelling catheter; SpO^2^, percutaneous arterial oxygen saturation.

Supplementary Table 8. The association of SHR and GV with 1-year mortality in NSTEMI patients.

| **Variables** | **Model 1** |  | **Model 2** |  | **Model 3** |  |
| --- | --- | --- | --- | --- | --- | --- |
|  | **HR 95%CI(c1-c2)** | ***P*** | **HR 95%CI(c1-c2)** | ***P*** | **HR 95%CI(c1-c2)** | ***P*** |
| **Overall** |  |  |  |  |  |  |
| SHR |  |  |  |  |  |  |
| T1(≤0.85) [Reference] | 1 |  | 1 |  | 1 |  |
| T2(0.85-1.11) | 0.92 (0.79-1.08) | 0.31 | 0.92 (0.78-1.07) | 0.277 | 1.03 (1.02-1.04) | ＜0.001 |
| T3(>1.11) | 1.12 (0.97-1.30) | 0.128 | 1.11 (0.96-1.29) | 0.166 | 0.97 (0.93-1.00) | 0.083 |
| P for trend | 1.06 (0.98-1.14) | 0.132 | 1.06 (0.98-1.14) | 0.163 | 1.06 (0.95-1.18) | 0.315 |
| GluCV |  |  |  |  |  |  |
| T1(≤24.88) [Reference] | 1 |  | 1 |  | 1 |  |
| T2(24.88-37.61) | 1.66 (1.40-1.97) | ＜0.001 | 1.81 (1.52-2.15) | ＜0.001 | 1.03 (1.02-1.05) | ＜0.001 |
| T3(>37.61) | 2.37 (2.01-2.79) | ＜0.001 | 3.25 (2.73-3.88) | ＜0.001 | 0.87 (0.83-0.90) | ＜0.001 |
| P for trend | 1.52 (1.41-1.65) | ＜0.001 | 1.80 (1.65-1.96) | ＜0.001 | 1.44 (1.31-1.59) | ＜0.001 |
| **Patients with NGR** |  |  |  |  |  |  |
| SHR |  |  |  |  |  |  |
| T1(≤0.85) [Reference] | 1 |  | 1 |  | 1 |  |
| T2(0.85-1.11) | 0.99 (0.63-1.54) | 0.95 | 0.93 (0.59-1.45) | 0.745 | 1.02 (1.01-1.04) | 0.002 |
| T3(>1.11) | 0.87 (0.55-1.38) | 0.549 | 0.77 (0.48-1.24) | 0.28 | 0.37 (0.22-0.62) | ＜0.001 |
| P for trend | 0.92 (0.74-1.16) | 0.496 | 0.87 (0.69-1.10) | 0.24 | 0.86 (0.65-1.15) | 0.319 |
| GluCV |  |  |  |  |  |  |
| T1(≤24.88) [Reference] | 1 |  | 1 |  | 1 |  |
| T2(24.88-37.61) | 1.52 (1.02-2.26) | 0.041 | 1.64 (1.10-2.46) | 0.016 | 1.03 (1.01-1.04) | ＜0.001 |
| T3(>37.61) | 3.41 (2.28-5.09) | ＜0.001 | 3.54 (2.35-5.35) | ＜0.001 | 0.46 (0.28-0.75) | 0.002 |
| P for trend | 1.83 (1.49-2.25) | ＜0.001 | 1.87 (1.52-2.31) | ＜0.001 | 1.52 (1.20-1.92) | ＜0.001 |
| **Patients with Pre-DM** |  |  |  |  |  |  |
| SHR |  |  |  |  |  |  |
| T1(≤0.85) [Reference] | 1 |  | 1 |  | 1 |  |
| T2(0.85-1.11) | 0.65 (0.42-1.01) | 0.058 | 0.71 (0.46-1.10) | 0.121 | 1.03 (1.01-1.05) | ＜0.001 |
| T3(>1.11) | 1.35 (0.90-2.01) | 0.148 | 1.33 (0.89-1.99) | 0.167 | 1.63 (0.77-3.49) | 0.205 |
| P for trend | 1.14 (0.91-1.42) | 0.253 | 1.13 (0.91-1.41) | 0.256 | 0.91 (0.61-1.34) | 0.627 |
| GluCV |  |  |  |  |  |  |
| T1(≤24.88) [Reference] | 1 |  | 1 |  | 1 |  |
| T2(24.88-37.61) | 1.85 (1.22-2.80) | 0.004 | 1.54 (1.00-2.38) | 0.051 | 1.03 (1.02-1.05) | ＜0.001 |
| T3(>37.61) | 3.00 (1.92-4.68) | ＜0.001 | 3.13 (2.00-4.91) | ＜0.001 | 1.31 (0.60-2.86) | 0.5 |
| P for trend | 1.73 (1.39-2.16) | ＜0.001 | 1.77 (1.41-2.23) | ＜0.001 | 1.54 (1.16-2.03) | 0.002 |
| **Patients with DM** |  |  |  |  |  |  |
| SHR |  |  |  |  |  |  |
| T1(≤0.85) [Reference] | 1 |  | 1 |  | 1 |  |
| T2(0.85-1.11) | 0.93 (0.78-1.12) | 0.444 | 0.92 (0.76-1.11) | 0.376 | 1.03 (1.02-1.04) | ＜0.001 |
| T3(>1.11) | 1.14 (0.96-1.35) | 0.142 | 1.11 (0.94-1.32) | 0.223 | 0.98 (0.95-1.02) | 0.43 |
| P for trend | 1.07 (0.98-1.16) | 0.15 | 1.06 (0.97-1.15) | 0.226 | 1.09 (0.96-1.24) | 0.173 |
| GluCV |  |  |  |  |  |  |
| T1(≤24.88) [Reference] | 1 |  | 1 |  | 1 |  |
| T2(24.88-37.61) | 1.75 (1.41-2.19) | ＜0.001 | 1.90 (1.51-2.37) | ＜0.001 | 1.03 (1.02-1.04) | ＜0.001 |
| T3(>37.61) | 2.46 (2.00-3.03) | ＜0.001 | 3.25 (2.61-4.06) | ＜0.001 | 0.89 (0.85-0.93) | ＜0.001 |
| P for trend | 1.54 (1.40-1.69) | ＜0.001 | 1.79 (1.61-1.98) | ＜0.001 | 1.45 (1.29-1.64) | ＜0.001 |

Model 1 (unadjusted), Model 2 (adjusted for age and glycated hemoglobin [HbA1c]), and Model 3 (adjusted for all potential covariates), NGR, normal glucose regulation; Pre-DM, Pre-diabetes mellitus; DM, diabetes mellitus.

Supplementary Table 9. The association of SHR and GV with 1-year mortality in STEMI patients

| **Variables** | **Model 1** |  | **Model 2** |  | **Model 3** |  |
| --- | --- | --- | --- | --- | --- | --- |
|  | **HR** **95%CI(c1-c2)** | ***P*** | **HR 95%CI(c1-c2)** | ***P*** | **HR 95%CI(c1-c2)** | ***P*** |
| **Overall** |  |  |  |  |  |  |
| SHR |  |  |  |  |  |  |
| T1(≤0.92) [Reference] | 1 |  | 1 |  | 1 |  |
| T2(0.92-1.25) | 0.73 (0.53-1.01) | 0.056 | 0.74 (0.54-1.03) | 0.075 | 1.02 (1.01-1.04) | < 0.001 |
| T3(>1.25) | 0.80 (0.58-1.10) | 0.169 | 0.86 (0.63-1.18) | 0.351 | 1.06 (0.99-1.12) | 0.093 |
| P for trend | 0.89 (0.76-1.05) | 0.157 | 0.92 (0.78-1.09) | 0.326 | 0.98 (0.77-1.26) | 0.889 |
| GluCV |  |  |  |  |  |  |
| T1(≤22.96) [Reference] | 1 |  | 1 |  | 1 |  |
| T2(22.96-33.65) | 1.49 (1.04-2.12) | 0.028 | 1.46 (1.02-2.08) | 0.04 | 1.02 (1.01-1.04) | < 0.001 |
| T3(>33.65) | 1.93 (1.37-2.70) | < 0.001 | 1.94 (1.34-2.79) | < 0.001 | 1.01 (0.94-1.08) | 0.788 |
| P for trend | 1.38 (1.17-1.63) | < 0.001 | 1.39 (1.16-1.66) | < 0.001 | 1.05 (0.85-1.29) | 0.646 |
| **Patients with NGR** |  |  |  |  |  |  |
| SHR |  |  |  |  |  |  |
| T1(≤0.92) [Reference] | 1 |  | 1 |  | 1 |  |
| T2(0.92-1.25) | 1.53 (0.54-4.34) | 0.425 | 1.56 (0.54-4.48) | 0.409 | 1.04 (1.01-1.07) | 0.02 |
| T3(>1.25) | 1.39 (0.49-3.94) | 0.539 | 1.33 (0.44-4.00) | 0.617 | 0.94 (0.21-4.29) | 0.935 |
| P for trend | 1.13 (0.70-1.81) | 0.622 | 1.10 (0.66-1.83) | 0.716 | 1.51 (0.51-4.46) | 0.452 |
| GluCV |  |  |  |  |  |  |
| T1(≤22.96) [Reference] | 1 |  | 1 |  | 1 |  |
| T2(22.96-33.65) | 1.51 (0.69-3.28) | 0.303 | 1.38 (0.63-3.01) | 0.423 | 1.04 (1.00-1.07) | 0.031 |
| T3(>33.65) | 0.56 (0.16-1.95) | 0.366 | 0.57 (0.16-1.97) | 0.373 | 0.76 (0.18-3.18) | 0.708 |
| P for trend | 0.90 (0.56-1.45) | 0.664 | 0.89 (0.54-1.45) | 0.635 | 0.66 (0.34-1.29) | 0.226 |
| **Patients with pre-DM** |  |  |  |  |  |  |
| SHR |  |  |  |  |  |  |
| T1(≤0.92) [Reference] | 1 |  | 1 |  | 1 |  |
| T2(0.92-1.25) | 0.87 (0.33-2.28) | 0.774 | 0.79 (0.30-2.08) | 0.628 | 1.03 (0.99-1.06) | 0.116 |
| T3(>1.25) | 1.65 (0.68-3.96) | 0.265 | 1.97 (0.79-4.90) | 0.146 | 0.32 (0.04-2.40) | 0.267 |
| P for trend | 1.28 (0.81-2.04) | 0.293 | 1.37 (0.84-2.24) | 0.203 | 0.57 (0.18-1.82) | 0.345 |
| GluCV |  |  |  |  |  |  |
| T1(≤22.96) [Reference] | 1 |  | 1 |  | 1 |  |
| T2(22.96-33.65) | 1.49 (0.61-3.67) | 0.386 | 1.30 (0.52-3.26) | 0.581 | 1.02 (0.99-1.05) | 0.221 |
| T3(>33.65) | 2.28 (0.88-5.91) | 0.091 | 2.46 (0.95-6.42) | 0.065 | 0.36 (0.05-2.63) | 0.313 |
| P for trend | 1.51 (0.94-2.43) | 0.092 | 1.56 (0.95-2.56) | 0.078 | 1.35 (0.58-3.14) | 0.484 |
| **Patients with DM** |  |  |  |  |  |  |
| SHR |  |  |  |  |  |  |
| T1(≤0.92) [Reference] | 1 |  | 1 |  | 1 |  |
| T2(0.92-1.25) | 0.65 (0.45-0.94) | 0.021 | 0.65 (0.45-0.95) | 0.024 | 1.02 (1.01-1.04) | < 0.001 |
| T3(>1.25) | 0.67 (0.47-0.97) | 0.034 | 0.71 (0.49-1.03) | 0.071 | 1.04 (0.97-1.11) | 0.277 |
| P for trend | 0.81 (0.67-0.98) | 0.027 | 0.83 (0.69-1.01) | 0.057 | 0.94 (0.68-1.29) | 0.696 |
| GluCV |  |  |  |  |  |  |
| T1(≤22.96) [Reference] | 1 |  | 1 |  | 1 |  |
| T2(22.96-33.65) | 1.52 (0.96-2.41) | 0.071 | 1.58 (0.99-2.51) | 0.053 | 1.02 (1.01-1.04) | < 0.001 |
| T3(>33.65) | 2.12 (1.38-3.25) | 0.001 | 2.23 (1.41-3.54) | 0.001 | 0.99 (0.92-1.07) | 0.87 |
| P for trend | 1.44 (1.18-1.77) | < 0.001 | 1.48 (1.19-1.84) | < 0.001 | 1.04 (0.81-1.34) | 0.758 |

Model 1 (unadjusted), Model 2 (adjusted for age and glycated hemoglobin [HbA1c]), and Model 3 (adjusted for all potential covariates), NGR, normal glucose regulation; Pre-DM, Pre-diabetes mellitus; DM, diabetes mellitus.

Supplementary Table 10. The association of SHR and GV with 1-year mortality in NSTEMI patients. Full adjustment (including the frequency of blood sugar monitoring)

| **Variables** | **Model 1** |  | **Model 2** |  | **Model 3** |  |
| --- | --- | --- | --- | --- | --- | --- |
|  | **HR 95%CI(c1-c2)** | ***P*** | **HR 95%CI(c1-c2)** | ***P*** | **HR 95%CI(c1-c2)** | ***P*** |
| **Overall** |  |  |  |  |  |  |
| SHR |  |  |  |  |  |  |
| T1(≤0.85) [Reference] | 1 |  | 1 |  | 1 |  |
| T2(0.85-1.11) | 0.92 (0.79-1.08) | 0.31 | 0.92 (0.78-1.07) | 0.277 | 1.03 (1.02-1.04) | ＜0.001 |
| T3(>1.11) | 1.12 (0.97-1.30) | 0.128 | 1.11 (0.96-1.29) | 0.166 | 0.97 (0.93-1.00) | 0.083 |
| P for trend | 1.06 (0.98-1.14) | 0.132 | 1.06 (0.98-1.14) | 0.163 | NA | NA |
| GluCV |  |  |  |  |  |  |
| T1(≤24.88) [Reference] | 1 |  | 1 |  | 1 |  |
| T2(24.88-37.61) | 1.66 (1.40-1.97) | ＜0.001 | 1.81 (1.52-2.15) | ＜0.001 | 1.03 (1.02-1.05) | ＜0.001 |
| T3(>37.61) | 2.37 (2.01-2.79) | ＜0.001 | 3.25 (2.73-3.88) | ＜0.001 | 0.87 (0.83-0.90) | ＜0.001 |
| P for trend | 1.52 (1.41-1.65) | ＜0.001 | 1.80 (1.65-1.96) | ＜0.001 | NA | NA |
| **Patients with NGR** |  |  |  |  |  |  |
| SHR |  |  |  |  |  |  |
| T1(≤0.85) [Reference] | 1 |  | 1 |  | 1 |  |
| T2(0.85-1.11) | 0.99 (0.63-1.54) | 0.95 | 0.93 (0.59-1.45) | 0.745 | 1.02 (1.01-1.04) | 0.002 |
| T3(>1.11) | 0.87 (0.55-1.38) | 0.549 | 0.77 (0.48-1.24) | 0.28 | 0.37 (0.22-0.62) | ＜0.001 |
| P for trend | 0.92 (0.74-1.16) | 0.496 | 0.87 (0.69-1.10) | 0.24 | NA | NA |
| GluCV |  |  |  |  |  |  |
| T1(≤24.88) [Reference] | 1 |  | 1 |  | 1 |  |
| T2(24.88-37.61) | 1.52 (1.02-2.26) | 0.041 | 1.64 (1.10-2.46) | 0.016 | 1.03 (1.01-1.04) | ＜0.001 |
| T3(>37.61) | 3.41 (2.28-5.09) | ＜0.001 | 3.54 (2.35-5.35) | ＜0.001 | 0.46 (0.28-0.75) | 0.002 |
| P for trend | 1.83 (1.49-2.25) | ＜0.001 | 1.87 (1.52-2.31) | ＜0.001 | NA | NA |
| **Patients with Pre-DM** |  |  |  |  |  |  |
| SHR |  |  |  |  |  |  |
| T1(≤0.85) [Reference] | 1 |  | 1 |  | 1 |  |
| T2(0.85-1.11) | 0.65 (0.42-1.01) | 0.058 | 0.71 (0.46-1.10) | 0.121 | 1.03 (1.01-1.05) | ＜0.001 |
| T3(>1.11) | 1.35 (0.90-2.01) | 0.148 | 1.33 (0.89-1.99) | 0.167 | 1.63 (0.77-3.49) | 0.205 |
| P for trend | 1.14 (0.91-1.42) | 0.253 | 1.13 (0.91-1.41) | 0.256 | NA | NA |
| GluCV |  |  |  |  |  |  |
| T1(≤24.88) [Reference] | 1 |  | 1 |  | 1 |  |
| T2(24.88-37.61) | 1.85 (1.22-2.80) | 0.004 | 1.54 (1.00-2.38) | 0.051 | 1.03 (1.02-1.05) | ＜0.001 |
| T3(>37.61) | 3.00 (1.92-4.68) | ＜0.001 | 3.13 (2.00-4.91) | ＜0.001 | 1.31 (0.60-2.86) | 0.5 |
| P for trend | 1.73 (1.39-2.16) | ＜0.001 | 1.77 (1.41-2.23) | ＜0.001 | NA | NA |
| **Patients with DM** |  |  |  |  |  |  |
| SHR |  |  |  |  |  |  |
| T1(≤0.85) [Reference] | 1 |  | 1 |  | 1 |  |
| T2(0.85-1.11) | 0.93 (0.78-1.12) | 0.444 | 0.92 (0.76-1.11) | 0.376 | 1.03 (1.02-1.04) | ＜0.001 |
| T3(>1.11) | 1.14 (0.96-1.35) | 0.142 | 1.11 (0.94-1.32) | 0.223 | 0.98 (0.95-1.02) | 0.43 |
| P for trend | 1.07 (0.98-1.16) | 0.15 | 1.06 (0.97-1.15) | 0.226 | NA | NA |
| GluCV |  |  |  |  |  |  |
| T1(≤24.88) [Reference] | 1 |  | 1 |  | 1 |  |
| T2(24.88-37.61) | 1.75 (1.41-2.19) | ＜0.001 | 1.90 (1.51-2.37) | ＜0.001 | 1.03 (1.02-1.04) | ＜0.001 |
| T3(>37.61) | 2.46 (2.00-3.03) | ＜0.001 | 3.25 (2.61-4.06) | ＜0.001 | 0.89 (0.85-0.93) | ＜0.001 |
| P for trend | 1.54 (1.40-1.69) | ＜0.001 | 1.79 (1.61-1.98) | ＜0.001 | NA | NA |

Model 1 (unadjusted), Model 2 (adjusted for age and glycated hemoglobin [HbA1c]), and Model 3 (adjusted for all potential covariates), NGR, normal glucose regulation; Pre-DM, Pre-diabetes mellitus; DM, diabetes mellitus.

Supplementary Table 11. The association of SHR and GV with 1-year mortality in STEMI patients. Full adjustment (including the frequency of blood sugar monitoring)

| **Variables** | **Model 1** |  | **Model 2** |  | **Model 3** |  |
| --- | --- | --- | --- | --- | --- | --- |
|  | **HR 95%CI(c1-c2)** | ***P*** | **HR 95%CI(c1-c2)** | ***P*** | **HR 95%CI(c1-c2)** | ***P*** |
| **Overall** |  |  |  |  |  |  |
| SHR |  |  |  |  |  |  |
| T1(≤0.92) [Reference] | 1 |  | 1 |  | 1 |  |
| T2(0.92-1.25) | 0.73 (0.53-1.01) | 0.056 | 0.74 (0.54-1.03) | 0.075 | 1.02 (1.01-1.04) | < 0.001 |
| T3(>1.25) | 0.80 (0.58-1.10) | 0.169 | 0.86 (0.63-1.18) | 0.351 | 1.06 (0.99-1.12) | 0.093 |
| P for trend | 0.89 (0.76-1.05) | 0.157 | 0.92 (0.78-1.09) | 0.326 | NA | NA |
| GluCV |  |  |  |  |  |  |
| T1(≤22.96) [Reference] | 1 |  | 1 |  | 1 |  |
| T2(22.96-33.65) | 1.49 (1.04-2.12) | 0.028 | 1.46 (1.02-2.08) | 0.04 | 1.02 (1.01-1.04) | < 0.001 |
| T3(>33.65) | 1.93 (1.37-2.70) | < 0.001 | 1.94 (1.34-2.79) | < 0.001 | 1.01 (0.94-1.08) | 0.788 |
| P for trend | 1.38 (1.17-1.63) | < 0.001 | 1.39 (1.16-1.66) | < 0.001 | NA | NA |
| **Patients with NGR** |  |  |  |  |  |  |
| SHR |  |  |  |  |  |  |
| T1(≤0.92) [Reference] | 1 |  | 1 |  | 1 |  |
| T2(0.92-1.25) | 1.53 (0.54-4.34) | 0.425 | 1.56 (0.54-4.48) | 0.409 | 1.04 (1.01-1.07) | 0.02 |
| T3(>1.25) | 1.39 (0.49-3.94) | 0.539 | 1.33 (0.44-4.00) | 0.617 | 0.94 (0.21-4.29) | 0.935 |
| P for trend | 1.13 (0.70-1.81) | 0.622 | 1.10 (0.66-1.83) | 0.716 | NA | NA |
| GluCV |  |  |  |  |  |  |
| T1(≤22.96) [Reference] | 1 |  | 1 |  | 1 |  |
| T2(22.96-33.65) | 1.51 (0.69-3.28) | 0.303 | 1.38 (0.63-3.01) | 0.423 | 1.04 (1.00-1.07) | 0.031 |
| T3(>33.65) | 0.56 (0.16-1.95) | 0.366 | 0.57 (0.16-1.97) | 0.373 | 0.76 (0.18-3.18) | 0.708 |
| P for trend | 0.90 (0.56-1.45) | 0.664 | 0.89 (0.54-1.45) | 0.635 | NA | NA |
| **Patients with pre-DM** |  |  |  |  |  |  |
| SHR |  |  |  |  |  |  |
| T1(≤0.92) [Reference] | 1 |  | 1 |  | 1 |  |
| T2(0.92-1.25) | 0.87 (0.33-2.28) | 0.774 | 0.79 (0.30-2.08) | 0.628 | 1.03 (0.99-1.06) | 0.116 |
| T3(>1.25) | 1.65 (0.68-3.96) | 0.265 | 1.97 (0.79-4.90) | 0.146 | 0.32 (0.04-2.40) | 0.267 |
| P for trend | 1.28 (0.81-2.04) | 0.293 | 1.37 (0.84-2.24) | 0.203 | NA | NA |
| GluCV |  |  |  |  |  |  |
| T1(≤22.96) [Reference] | 1 |  | 1 |  | 1 |  |
| T2(22.96-33.65) | 1.49 (0.61-3.67) | 0.386 | 1.30 (0.52-3.26) | 0.581 | 1.02 (0.99-1.05) | 0.221 |
| T3(>33.65) | 2.28 (0.88-5.91) | 0.091 | 2.46 (0.95-6.42) | 0.065 | 0.36 (0.05-2.63) | 0.313 |
| P for trend | 1.51 (0.94-2.43) | 0.092 | 1.56 (0.95-2.56) | 0.078 | NA | NA |
| **Patients with DM** |  |  |  |  |  |  |
| SHR |  |  |  |  |  |  |
| T1(≤0.92) [Reference] | 1 |  | 1 |  | 1 |  |
| T2(0.92-1.25) | 0.65 (0.45-0.94) | 0.021 | 0.65 (0.45-0.95) | 0.024 | 1.02 (1.01-1.04) | < 0.001 |
| T3(>1.25) | 0.67 (0.47-0.97) | 0.034 | 0.71 (0.49-1.03) | 0.071 | 1.04 (0.97-1.11) | 0.277 |
| P for trend | 0.81 (0.67-0.98) | 0.027 | 0.83 (0.69-1.01) | 0.057 | NA | NA |
| GluCV |  |  |  |  |  |  |
| T1(≤22.96) [Reference] | 1 |  | 1 |  | 1 |  |
| T2(22.96-33.65) | 1.52 (0.96-2.41) | 0.071 | 1.58 (0.99-2.51) | 0.053 | 1.02 (1.01-1.04) | < 0.001 |
| T3(>33.65) | 2.12 (1.38-3.25) | 0.001 | 2.23 (1.41-3.54) | 0.001 | 0.99 (0.92-1.07) | 0.87 |
| P for trend | 1.44 (1.18-1.77) | < 0.001 | 1.48 (1.19-1.84) | < 0.001 | NA | NA |

Model 1 (unadjusted), Model 2 (adjusted for age and glycated hemoglobin [HbA1c]), and Model 3 (adjusted for all potential covariates), NGR, normal glucose regulation; Pre-DM, Pre-diabetes mellitus; DM, diabetes mellitus.

Supplementary Table 12. The association of the combination of SHR and GV with all-cause mortality in STEMI patients.

| **Variables** | **Model 1** | |  | | **Model 2** | |  | | **Model 3** | |  |  |
| --- | --- | --- | --- | --- | --- | --- | --- | --- | --- | --- | --- | --- |
|  | **HR** **95%CI(c1-c2)** | | ***P*** | | **HR 95%CI(c1-c2)** | | ***P*** | | **HR 95%CI(c1-c2)** | | ***P*** |  |
| **Overall** |  | |  | |  | |  | |  | |  |  |
| Group 1 [Reference] | 1 | |  | | 1 | |  | | 1 | |  |  |
| Group 2 | 0.77 (0.44-1.34) | | 0.35 | | 0.77 (0.44-1.34) | | 0.349 | | 1.02 (1.01-1.03) | | < 0.001 |  |
| Group 3 | 1.74 (1.06-2.84) | | 0.028 | | 1.66 (1.00-2.75) | | 0.049 | | 1.02 (0.95-1.09) | | 0.606 |  |
| Group 4 | 1.31 (0.82-2.09) | | 0.261 | | 1.30 (0.81-2.08) | | 0.281 | | 0.94 (0.51-1.75) | | 0.851 |  |
| P for trend | 1.15 (1.01-1.31) | | 0.03 | | 1.14 (0.99-1.30) | | 0.06 | | 1.03 (0.86-1.23) | | 0.764 |  |
| **Patients with NGR** |  | |  | |  | |  | |  | |  |  |
| Group 1 [Reference] | 1 | |  | | 1 | |  | | 1 | |  |  |
| Group 2 | 1.51 (0.48-4.74) | | 0.481 | | 1.31 (0.40-4.25) | | 0.651 | | 1.04 (1.01-1.07) | | 0.019 |  |
| Group 3 | 1.20 (0.13-10.71) | | 0.872 | | 0.70 (0.08-6.48) | | 0.757 | | 1.02 (0.23-4.46) | | 0.981 |  |
| Group 4 | 1.50 (0.49-4.60) | | 0.478 | | 1.37 (0.43-4.30) | | 0.594 | | 3.29 (0.42-26.08) | | 0.259 |  |
| P for trend | 1.08 (0.80-1.47) | | 0.616 | | 1.06 (0.78-1.46) | | 0.704 | | 1.66 (0.70-3.91) | | 0.246 |  |
| **Patients with Pre-DM** |  | |  | |  | |  | |  | |  |  |
| Group 1 [Reference] | 1 | |  | | 1 | |  | | 1 | |  |  |
| Group 2 | 0.21 (0.04-1.03) | | 0.055 | | 0.21 (0.04-0.99) | | 0.049 | | 1.02 (0.99-1.05) | | 0.255 |  |
| Group 3 | 0.40 (0.10-1.56) | | 0.188 | | 0.36 (0.09-1.41) | | 0.142 | | 0.37 (0.05-2.70) | | 0.328 |  |
| Group 4 | 1.35 (0.55-3.32) | | 0.51 | | 1.26 (0.51-3.12) | | 0.618 | | 0.37 (0.04-3.16) | | 0.366 |  |
| P for trend | 1.29 (0.91-1.84) | | 0.152 | | 1.27 (0.89-1.81) | | 0.185 | | 0.70 (0.28-1.73) | | 0.435 |  |
| **Patients with DM** |  | |  | |  | |  | |  | |  |  |
| Group 1 [Reference] | 1 | |  | | 1 | |  | | 1 | |  |  |
| Group 2 | 0.80 (0.37-1.73) | | 0.566 | | 0.82 (0.38-1.78) | | 0.62 | | 1.02 (1.01-1.04) | | < 0.001 |  |
| Group 3 | 2.06 (1.06-4.02) | | 0.034 | | 2.17 (1.10-4.29) | | 0.025 | | 1.00 (0.93-1.08) | | 0.976 |  |
| Group 4 | 1.33 (0.69-2.58) | | 0.391 | | 1.41 (0.72-2.73) | | 0.314 | | 0.90 (0.38-2.12) | | 0.805 |  |
| P for trend | 1.12 (0.96-1.32) | | 0.158 | | 1.12 (0.95-1.32) | | 0.194 | | 1.06 (0.84-1.34) | | 0.634 |  |

Group 1: Low SHR and Low GluCV (SHR < 0.92 and GluCV < 22.96); Group 2: Low SHR and High GluCV (SHR < 0.92 and GluCV > 33.65); Group 3: High SHR and Low GluCV (SHR > 1.25 and GluCV < 22.96); Group 4: High SHR and High GluCV (SHR > 1.25 and GluCV > 33.65), NGR, normal glucose regulation; Pre-DM, Pre-diabetes mellitus; DM, diabetes mellitus.

Supplementary Table 13. The results of the proportional hazards test (Cox model) for the NGR group proportion in patients with NSTEMI.

| **Variable** | ***P*** | |
| --- | --- | --- |
| joint_group | 0.092 | |
| HbA1C | 0.183 | |
| Glucose | 0.544 | |
| SHR | 0.951 | |
| Weight | 0.228 | |
| BMI | 0.817 | |
| Age | 0.019 | |
| CHF | 0.035 | |
| Chronic_pulmonary_disease | 0.016 | |
| Renal_disease | ＜0.001 | |
| APSIII | 0.171 | |
| GCS | 0.901 | |
| Invasion | 0.829 | |
| SAPSII | 0.017 | |
| Ventilation | 0.372 | |
| Alb | 0.642 | |
| BUN | ＜0.001 | |
| CK-MB | 0.244 | |
| Platelet | 0.643 | |
| WBC | 0.041 | |
| Intervention | 0.134 | |
| RBC | 0.319 | |
| HeartRate | 0.125 | |
| RR | 0.485 | |
| Temperature | 0.426 | |
| SpO^2^ | 0.78 | |
| GLOBAL | ＜0.001 | |

BMI, body mass index; HR, heart rate; RR, Respiratory Rate; CHF, congestive heart failure; BUN, blood urea nitrogen; HbA1c, glycated hemoglobin; RBC, red blood cell; GluCV, Blood glucose coefficient of variation; SHR, Stress hyperglycemia ratio; Alb, albumin; CK-MB, creatine kinase-MB; WBC, white blood cell; GCS, Glasgow Coma Scale; APSIII, Acute Physiology Score III; SAPSII, Simplified Acute Physiology Score II; Intervention, hypoglycemic drugs and insulin therapy; Invasion, intravascular indwelling catheter; SpO^2^, percutaneous arterial oxygen saturation.

Supplementary Table 14. The results of the proportional hazards test (Cox model) for the Pre-DM group proportion in patients with NSTEMI.

| **Variable** | ***P*** | |
| --- | --- | --- |
| joint_group | 0.105 | |
| HbA1C | 0.545 | |
| Glucose | 0.715 | |
| SHR | 0.629 | |
| Weight | 0.006 | |
| BMI | 0.349 | |
| Age | 0.986 | |
| CHF | 0.062 | |
| Chronic_pulmonary_disease | 0.421 | |
| Renal_disease | 0.143 | |
| APSIII | 0.245 | |
| GCS | 0.364 | |
| Invasion | 0.214 | |
| SAPSII | 0.224 | |
| Ventilation | 0.218 | |
| Alb | 0.112 | |
| BUN | 0.146 | |
| CKMB | 0.238 | |
| Platelet | 0.136 | |
| WBC | 0.207 | |
| Intervention | 0.023 | |
| RBC | 0.001 | |
| HeartRate | 0.984 | |
| RR | 0.804 | |
| Temperature | 0.197 | |
| SpO^2^ | 0.898 | |
| GLOBAL | 0.001 | |

BMI, body mass index; HR, heart rate; RR, Respiratory Rate; CHF, congestive heart failure; BUN, blood urea nitrogen; HbA1c, glycated hemoglobin; RBC, red blood cell; GluCV, Blood glucose coefficient of variation; SHR, Stress hyperglycemia ratio; Alb, albumin; CK-MB, creatine kinase-MB; WBC, white blood cell; GCS, Glasgow Coma Scale; APSIII, Acute Physiology Score III; SAPSII, Simplified Acute Physiology Score II; Intervention, hypoglycemic drugs and insulin therapy; Invasion, intravascular indwelling catheter; SpO^2^, percutaneous arterial oxygen saturation.

Supplementary Table 15. The results of the proportional hazards test (Cox model) for the DM group proportion in patients with NSTEMI.

| **Variable** | ***P*** | |
| --- | --- | --- |
| joint_group | 0.021 | |
| HbA1C | 0.528 | |
| Glucose | 0.119 | |
| SHR | 0.263 | |
| Weight | 0.587 | |
| BMI | 0.864 | |
| Age | 0.016 | |
| CHF | 0.255 | |
| Chronic_pulmonary_disease | 0.496 | |
| Renal_disease | 0.001 | |
| APSIII | 0.041 | |
| GCS | 0.317 | |
| Invasion | 0.309 | |
| SAPSII | 0.011 | |
| Ventilation | 0.53 | |
| Alb | 0.906 | |
| BUN | ＜0.001 | |
| CKMB | 0.01 | |
| Platelet | 0.011 | |
| WBC | 0.014 | |
| Intervention | 0.246 | |
| RBC | 0.218 | |
| HeartRate | 0.108 | |
| RR | 0.688 | |
| Temperature | 0.333 | |
| SpO^2^ | 0.551 | |
| GLOBAL | ＜0.001 | |

BMI, body mass index; HR, heart rate; RR, Respiratory Rate; CHF, congestive heart failure; BUN, blood urea nitrogen; HbA1c, glycated hemoglobin; RBC, red blood cell; GluCV, Blood glucose coefficient of variation; SHR, Stress hyperglycemia ratio; Alb, albumin; CK-MB, creatine kinase-MB; WBC, white blood cell; GCS, Glasgow Coma Scale; APSIII, Acute Physiology Score III; SAPSII, Simplified Acute Physiology Score II; Intervention, hypoglycemic drugs and insulin therapy; Invasion, intravascular indwelling catheter; SpO^2^, percutaneous arterial oxygen saturation.

Supplementary Table 16. The results of the proportional hazards test (Cox model) for the NGR group proportion in patients with STEMI.

| **Variable** | ***P*** | |
| --- | --- | --- |
| joint_group | 0.758 | |
| HbA1C | 0.347 | |
| Glucose | 0.388 | |
| SHR | 0.501 | |
| Weight | 0.035 | |
| BMI | 0.065 | |
| Age | 0.202 | |
| CHF | 0.272 | |
| Chronic_pulmonary_disease | 0.166 | |
| Renal_disease | 0.069 | |
| APSIII | 0.404 | |
| GCS | 0.831 | |
| Invasion | 0.513 | |
| SAPSII | 0.526 | |
| Ventilation | 0.942 | |
| Alb | 0.767 | |
| BUN | 0.206 | |
| CKMB | 0.245 | |
| Platelet | 0.335 | |
| WBC | 0.589 | |
| Intervention | 0.828 | |
| RBC | 0.549 | |
| HeartRate | 0.65 | |
| RR | 0.782 | |
| Temperature | 0.368 | |
| SpO^2^ | 0.188 | |
| GLOBAL | 0.156 | |

BMI, body mass index; HR, heart rate; RR, Respiratory Rate; CHF, congestive heart failure; BUN, blood urea nitrogen; HbA1c, glycated hemoglobin; RBC, red blood cell; GluCV, Blood glucose coefficient of variation; SHR, Stress hyperglycemia ratio; Alb, albumin; CK-MB, creatine kinase-MB; WBC, white blood cell; GCS, Glasgow Coma Scale; APSIII, Acute Physiology Score III; SAPSII, Simplified Acute Physiology Score II; Intervention, hypoglycemic drugs and insulin therapy; Invasion, intravascular indwelling catheter; SpO^2^, percutaneous arterial oxygen saturation.

Supplementary Table 17. The results of the proportional hazards test (Cox model) for the Pre-DM group proportion in patients with STEMI.

| **Variable** | ***P*** | |
| --- | --- | --- |
| joint_group | 0.044 | |
| HbA1C | 0.176 | |
| Glucose | 0.922 | |
| SHR | 0.945 | |
| Weight | 0.069 | |
| BMI | 0.031 | |
| Age | 0.141 | |
| CHF | 0.185 | |
| Chronic_pulmonary_disease | 0.526 | |
| Renal_disease | 0.001 | |
| APSIII | 0.936 | |
| GCS | 0.777 | |
| Invasion | 0.678 | |
| SAPSII | 0.648 | |
| Ventilation | 0.598 | |
| Alb | 0.414 | |
| BUN | 0.226 | |
| CKMB | 0.189 | |
| Platelet | 0.049 | |
| WBC | 0.002 | |
| Intervention | 0.216 | |
| RBC | 0.02 | |
| HeartRate | 0.347 | |
| RR | 0.203 | |
| Temperature | 0.859 | |
| SpO^2^ | 0.508 | |
| GLOBAL | 0.005 | |

BMI, body mass index; HR, heart rate; RR, Respiratory Rate; CHF, congestive heart failure; BUN, blood urea nitrogen; HbA1c, glycated hemoglobin; RBC, red blood cell; GluCV, Blood glucose coefficient of variation; SHR, Stress hyperglycemia ratio; Alb, albumin; CK-MB, creatine kinase-MB; WBC, white blood cell; GCS, Glasgow Coma Scale; APSIII, Acute Physiology Score III; SAPSII, Simplified Acute Physiology Score II; Intervention, hypoglycemic drugs and insulin therapy; Invasion, intravascular indwelling catheter; SpO^2^, percutaneous arterial oxygen saturation.

Supplementary Table 18. Discrimination of each predictive model for outcomes in the NSTEMI group.

| **Models** | **AUC** **95%CI(c1-c2)** | **Sensitivity** | **Specificity** | ***P*** |
| --- | --- | --- | --- | --- |
| **NGR** |  |  |  |  |
| GluCV_SHR | 0.667 (0.611-0.723) | 0.730 | 0.565 | 1.00 (Reference) |
| GluCV | 0.668 (0.613-0.724) | 0.730 | 0.574 | 0.102 |
| SHR | 0.489 (0.432-0.546) | 0.936 | 0.124 | ＜0.001 |
| APSIII | 0.669 (0.620-0.718) | 0.844 | 0.471 | 0.946 |
| SAPSII | 0.582 (0.527-0.638) | 0.624 | 0.523 | 0.034 |
| **Pre-DM** |  |  |  |  |
| GluCV_SHR | 0.646 (0.590-0.703) | 0.719 | 0.510 | 1.00 (Reference) |
| GluCV | 0.647 (0.591-0.703) | 0.719 | 0.516 | 0.606 |
| SHR | 0.525 (0.461-0.589) | 0.367 | 0.776 | 0.004 |
| APSIII | 0.677 (0.623-0.730) | 0.758 | 0.569 | 0.379 |
| SAPSII | 0.638 (0.581-0.696) | 0.664 | 0.576 | 0.841 |
| **DM** |  |  |  |  |
| GluCV_SHR | 0.615 (0.592-0.638) | 0.632 | 0.567 | 1.00 (Reference) |
| GluCV | 0.615 (0.593-0.638) | 0.555 | 0.643 | 0.260 |
| SHR | 0.506 (0.481-0.531) | 0.396 | 0.658 | ＜0.001 |
| APSIII | 0.671 (0.649-0.693) | 0.662 | 0.617 | ＜0.001 |
| SAPSII | 0.632 (0.609-0.656) | 0.530 | 0.671 | 0.307 |

NGR, Normal glucose regulation; Pre-DM, Pre-diabetes mellitus; DM, Diabetes mellitus; GluCV, Blood glucose coefficient of variation; SHR, Stress hyperglycemia ratio; APSIII, Acute Physiology Score III; SAPSII, Simplified Acute Physiology Score II.

Supplementary Table 19. Discrimination of each predictive model for outcomes in the STEMI group

| **Models** | **AUC** **95%CI (c1-c2)** | **Sensitivity** | **Specificity** | ***P*** |
| --- | --- | --- | --- | --- |
| **NGR** |  |  |  |  |
| GluCV_SHR | 0.564 (0.463-0.666) | 0.966 | 0.333 | 1.00 (Reference) |
| GluCV | 0.562 (0.460-0.664) | 0.966 | 0.333 | 0.738 |
| SHR | 0.568 (0.451-0.685) | 0.621 | 0.556 | 0.956 |
| APSIII | 0.773 (0.672-0.873) | 0.793 | 0.727 | 0.002 |
| SAPSII | 0.780 (0.683-0.878) | 0.793 | 0.737 | 0.002 |
| **Pre-DM** |  |  |  |  |
| GluCV_SHR | 0.638 (0.527-0.749) | 0.926 | 0.422 | 1.00 (Reference) |
| GluCV | 0.637 (0.526-0.747) | 0.926 | 0.422 | 0.755 |
| SHR | 0.620 (0.504-0.735) | 0.852 | 0.433 | 0.785 |
| APSIII | 0.763 (0.655-0.871) | 0.741 | 0.744 | 0.092 |
| SAPSII | 0.750 (0.644-0.856) | 0.630 | 0.789 | 0.139 |
| **DM** |  |  |  |  |
| GluCV_SHR | 0.617 (0.568-0.667) | 0.630 | 0.598 | 1.00 (Reference) |
| GluCV | 0.620 (0.570-0.669) | 0.630 | 0.598 | 0.083 |
| SHR | 0.432 (0.378-0.486) | 0.067 | 0.935 | ＜0.001 |
| APSIII | 0.730 (0.685-0.776) | 0.745 | 0.617 | ＜0.001 |
| SAPSII | 0.735 (0.689-0.781) | 0.764 | 0.612 | 0.001 |

NGR, normal glucose regulation; Pre-DM, Pre-diabetes mellitus; DM, diabetes mellitus; GluCV, Blood glucose coefficient of variation; SHR, Stress hyperglycemia ratio; APSIII, Acute Physiology Score III; SAPSII, Simplified Acute Physiology Score II.
Supplementary Table 20. The results of the proportional hazards test (Cox model) for the DM group proportion in patients with STEMI.

| **Variable** | ***P*** |
| --- | --- |
| joint_group | 0.116 |
| HbA1C | 0.142 |
| Glucose | 0.003 |
| SHR | 0.02 |
| Weight | ＜0.001 |
| BMI | ＜0.001 |
| Age | 0.729 |
| CHF | 0.167 |
| Chronic_pulmonary_disease | 0.753 |
| Renal_disease | 0.11 |
| APSIII | 0.094 |
| GCS | 0.357 |
| Invasion | 0.25 |
| SAPSII | 0.032 |
| Ventilation | 0.239 |
| Alb | 0.745 |
| BUN | 0.06 |
| CKMB | 0.011 |
| Platelet | 0.232 |
| WBC | 0.007 |
| Intervention | 0.283 |
| RBC | ＜0.001 |
| HeartRate | 0.359 |
| RR | 0.31 |
| Temperature | 0.489 |
| SpO^2^ | 0.668 |
| GLOBAL | ＜0.001 |

BMI, body mass index; HR, heart rate; RR, Respiratory Rate; CHF, congestive heart failure; BUN, blood urea nitrogen; HbA1c, glycated hemoglobin; RBC, red blood cell; GluCV, Blood glucose coefficient of variation; SHR, Stress hyperglycemia ratio; Alb, albumin; CK-MB, creatine kinase-MB; WBC, white blood cell; GCS, Glasgow Coma Scale; APSIII, Acute Physiology Score III; SAPSII, Simplified Acute Physiology Score II; Intervention, hypoglycemic drugs and insulin therapy; Invasion, intravascular indwelling catheter; SpO^2^, percutaneous arterial oxygen saturation.

Supplementary Fig. 1 Proportion of missing data values.

Supplementary Fig. 2 Lasso regression for variable selection. A. Variation patterns of feature coefficients; B. The process of determining the optimal value of λ in the Lasso regression model utilizes cross-validation.

**A**

**B**

Supplementary Fig. 3 Subgroup analysis of GluCV in NSTEMI patients

Supplementary Fig. 4 The Kaplan-Meier curves for the 1-year mortality of SHR, GV, and their combinations in STEMI patients. A. GluCV of NGR, B. SHR of NGR, C. GluCV+SHR of NGR, D. GluCV of Pre-DM, E. SHR of Pre-DM, F. GluCV+SHR of Pre-DM, G. GluCV of DM, H. SHR of DM, I. GluCV+SHR of DM. [A, D, G. T1 GluCV < 22.96; T2 22.96≤GluCV≤33.65; T3 GluCV >33.65. B, E, H. T1 SHR<0.92; T2 0.92≤SHR≤1.25; T3 SHR>1.25. C, F, I. Group 1: Low SHR and Low GluCV (SHR < 0.92 and GluCV < 22.96); Group 2: Low SHR and High GluCV (SHR < 0.92 and GluCV >33.65); Group 3: High SHR and Low GluCV (SHR > 1.25 and GluCV <  22.96); Group 4: High SHR and High GluCV (SHR > 1.25 and GluCV >33.65)].

Supplementary Fig. 5 Subgroup analysis of GluCV in STEMI patients

Supplementary Fig. 6 Subgroup analysis of SHR in NSTEMI patients

Supplementary Fig. 7 Subgroup analysis of SHR in STEMI patients

Supplementary Fig. 8 Trend analysis of DM with NSTEMI patients based on the proportional hazards assumption of the COX regression model.

Supplementary Fig. 9 Trend analysis of Pre-DM with STEMI patients based on the proportional hazards assumption of the COX regression model.

Supplementary Fig. 10 Landmark survival analysis of 1-year mortality with combined SHR and GluCV assessment in the Pre-DM with STEMI patients.

Supplementary Fig. 11 The Boruta algorithm ranks the importance of potential risk factors for 1-year mortality in the NGR group of patients with NSTEMI.

Supplementary Fig. 12 The Boruta algorithm ranks the importance of potential risk factors for 1-year mortality in the Pre-DM group of patients with NSTEMI.

Supplementary Fig. 13 The Boruta algorithm ranks the importance of potential risk factors for 1-year mortality in the DM group of patients with NSTEMI.

Supplementary Fig. 14 The Boruta algorithm ranks the importance of potential risk factors for 1-year mortality in NSTEMI patients. The x-axis delineates parameter nomenclature, whereas the y-axis quantifies standardized scores (Z-scores) across variables. Boxplot distributions graphically depict the dispersion characteristics of normalized values during model computation cycles. Green is an important variable, red is an unimportant variable, blue is a shadow variable, and yellow is a Tentative variable.

1. Preprocessing of Categorical Variables

All categorical variables in the datasets (NSTEMI-NGR, NSTEMI-Pre-DM, NSTEMI-DM) were processed using **one-hot encoding** (dummy variables) to avoid ordinal bias. Variables with binary categories (e.g., sex, mechanical ventilation status) were directly converted to 0/1 numeric values; multi-class categorical variables (e.g., race, comorbidity subtypes) were encoded into mutually exclusive binary features. Missing values were handled with **multiple imputation** (for variables with <20% missing values) or exclusion (for variables with ≥20% missing values), consistent with the preprocessing steps described in the main manuscript.

2. Hyperparameter Tuning Strategies for Each ML Algorithm

Hyperparameter optimization was performed for each algorithm to minimize overfitting and maximize predictive performance, with **10-fold stratified cross-validation** used to evaluate hyperparameter combinations in all tuning processes. The specific strategies for each model are as follows:

- **Random Forest (RF)**: Two complementary methods were used to determine the optimal mtry (number of variables sampled for splitting): (1) a grid search with mtry incremented by 5 (from 5 to the total number of features); (2) the train function in the caret package with **repeated 5-fold cross-validation (10 repeats)** and random search. The optimal ntree (number of trees) was set to 500 (verified by error convergence in the RF error curve). Other parameters were set to default values (e.g., nodesize = 1, proximity = TRUE).
- **XGBoost**: **Bayesian optimization** (package rBayesianOptimization) was used to tune key hyperparameters, with the search space: eta (0.01, 0.05, 0.1, 0.3), max_depth (6, 8, 12), min_child_weight (1, 10), subsample (0.5, 0.8, 1). The objective function was set to binary:logistic with eval_metric = "auc", and **early stopping (5 rounds)** was used to avoid overfitting with a maximum of 1000 iterations (nround). Fixed parameters included colsample_bytree = 0.6, lambda = 1, alpha = 0.
- **Support Vector Machine (SVM)**: **Grid search** (function tune.svm in package e1071) was performed for hyperparameter tuning, with the search space: gamma (10^(-6) to 10^1) and cost (10^(-10) to 10^10). The radial basis function (RBF) was used as the kernel, and probability = TRUE was enabled to output predictive probabilities for ROC curve analysis.
- **LightGBM**: A **grid search** was conducted for key hyperparameters: max_depth = 8, lambda_l1 (0–5), lambda_l2 (0–5), min_sum_hessian_in_leaf (0–2), feature_fraction (0.8, 1). The objective was set to binary with metric = "auc", 1000 maximum iterations, and early stopping (5 rounds). The final model used the parameter combination with the lowest L2 loss.
- **AdaBoost**: The optimal number of base classifiers (mfinal) was determined by **error curve iteration** (1 to 100 iterations), selecting the value with the minimum out-of-bag (OOB) classification error (final mfinal = 10 for all datasets). The base classifier was a decision tree, with boos = TRUE (bootstrap sampling for training data).

3. Mitigation of Class Imbalance

Class distribution in the mortality outcome (0 = survival, 1 = death) was first assessed for all datasets; no severe class imbalance (imbalance ratio < 4:1) was identified. To further ensure robust model performance for the minority class (death), we implemented two key strategies: (1) **stratified sampling** in all cross-validation and bootstrap procedures to preserve the original class ratio in training and test sets; (2) for tree-based models (RF, XGBoost, LightGBM), we set is_unbalance = FALSE (no artificial weight adjustment) to avoid overfitting to the minority class, as the mild imbalance was sufficiently addressed by stratified sampling.

4. Rigorous Model Validation Protocols

A two-tier validation strategy was used to comprehensively evaluate model performance and generalizability, with **a fixed random seed (1234)** set for all random processes to ensure full reproducibility:

1. **Bootstrap Validation (500 iterations)**: For each algorithm, 500 rounds of **stratified random sampling with replacement** were performed on the original dataset (sample size consistent with the original). For each bootstrap sample, the model was retrained with optimal hyperparameters, and predictive performance was evaluated on the **original full dataset**. Key metrics (AUC, sensitivity, specificity) and ROC curve data were recorded for each iteration, and the final performance was reported as the **mean ± standard deviation (SD)** of AUC values, with a 95% confidence interval (CI) calculated from the 5th and 95th percentiles of the bootstrap AUC distribution.
2. **Repeated Stratified 5-Fold Cross-Validation (100 repeats, 500 total folds)**: The dataset was split into 5 stratified folds (preserving class ratio) for 100 independent repeats, resulting in 500 total validation folds. For each fold, the model was trained on 4 folds and tested on the remaining 1 fold; predictive probabilities and ROC curves were recorded for each test set. The final performance was summarized as the mean AUC ± SD across all 500 folds, with a 95% CI from the 5th and 95th percentiles of the cross-validation AUC distribution.

5. Additional Reproducibility Details: All ML analyses were performed in **R software (version 4.5.1)**, with the following key packages used: randomForest (RF), xgboost (XGBoost), e1071 (SVM), lightgbm (LightGBM), adabag (AdaBoost), caret (cross-validation and tuning), pROC (ROC curve analysis), rBayesianOptimization (XGBoost tuning), and shapviz (SHAP value calculation for feature importance).
